# Binding‐Mediated Formation of Ribonucleoprotein Corona for Efficient Delivery and Control of CRISPR/Cas9

**DOI:** 10.1002/anie.202014162

**Published:** 2021-03-18

**Authors:** Jinjun Wu, Hanyong Peng, Xiufen Lu, Maode Lai, Hongquan Zhang, X. Chris Le

**Affiliations:** ^1^ Division of Analytical and Environmental Toxicology Department of Laboratory Medicine and Pathology Faculty of Medicine and Dentistry University of Alberta Edmonton Alberta T6G 2G3 Canada; ^2^ Department of Pathology Zhejiang University School of Medicine Hangzhou Zhejiang 310058 China

**Keywords:** CRISPR/Cas9, gene editing, gold nanoparticles, protein corona, protein delivery

## Abstract

Protein coronae formed with nanoparticles confer several useful properties. However, the non‐specific nature of protein corona formation makes it difficult to deliver specific proteins for therapeutic applications. Herein, we report on the construction of a new type of protein corona, termed binding‐mediated protein corona. This new corona enables the efficient and controllable delivery of functional proteins, which is otherwise challenging for conventional protein coronae. We show the design and delivery of the ribonucleoprotein corona for the CRISPR/Cas9 system. Successful gene editing in human cell lines (Hela and HEK293) demonstrates the efficient delivery, high stability, low cytotoxicity, and well‐controlled activity of the Cas9‐guide RNA ribonucleoprotein. The binding‐mediated protein corona strategy opens up new opportunities for therapeutic protein delivery.

## Graphical abstract

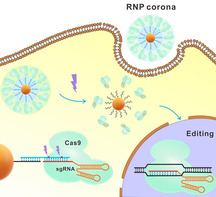

Nanoparticles (NPs) are known to adsorb proteins non‐specifically, forming “protein coronae”.^[1]^ The formation of a protein corona changes physicochemical and biological properties of NPs,^[2]^ such as decreasing cytotoxicity,^[3]^ altering biodistribution,^[4]^ increasing circulation time,^[5]^ enhancing cell‐specific targeting,^[6]^ and modifying cellular uptake.^[7]^ Thus, protein coronae can improve efficacy of drug delivery when used as carriers to deliver therapeutic molecules.[^6a^, ^8^]

However, protein coronae have only been applied to the delivery of functional small molecules and oligonucleotides based on their electrostatic or hydrophobic interactions with the proteins. Protein coronae have not yet been used for targeted delivery of therapeutic proteins, because of the following challenges. Proteins can irreversibly bind to the nanoparticle surface, leading to changes of protein conformation and loss of biological functions, when the proteins are encapsulated in the inner layer of the corona.^[9]^ On the other hand, when the proteins of interest (payload proteins) are loaded on the outer layer of the protein corona, these payload proteins can be replaced by abundant serum proteins and readily dissociate from the corona, resulting in the leakage problem.^[10]^

Here, we describe an affinity binding strategy to construct a new type of protein corona, termed binding‐mediated protein corona (BMPC) (Scheme 1). The BMPC innovation uses affinity ligands pre‐conjugated onto NPs to assemble specific payload proteins into a uniform monolayer and serve in a spatial role to avoid irreversible binding of the payload proteins directly with the core of the NPs. Additionally, multiple affinity molecules within a nanosized surface offers polyvalent interactions between the payload proteins and the affinity ligands, minimizing leakage of the payload proteins. Thus, the binding‐mediated protein corona overcomes both limitations of the traditional protein corona for delivery of biologically active proteins: irreversible binding and protein leakage.

**Scheme 1:**
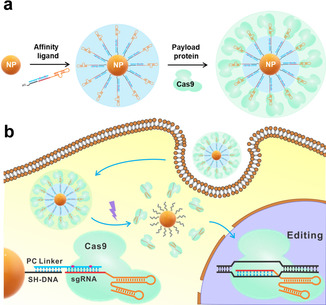
Schematic illustration of a) the Cas9‐sgRNA ribonucleoprotein corona and b) light‐initiated release and activation of the ribonucleoprotein inside human cells. A photo‐cleavable linker (PC linker, blue), consisting of two cleavage sites, is hybridized with a sgRNA (red) and a thiol‐labeled short DNA (SH‐DNA, black), resulting in an assembly of the sgRNA on nanoparticles (NPs). The Cas9 protein interacts with the sgRNA to form a ribonucleoprotein (RNP) corona. The binding‐mediated ribonucleoprotein corona enters the cells without requiring transfection reagents. Light (365 nm) breaks the photocleavable linker, releasing the RNP inside the cells. The released RNP enters the nucleus and performs gene editing.

We chose to deliver the Cas9 ribonucleoprotein (RNP) as an example, consisting of the Cas9 protein and a specific single‐guide RNA (sgRNA), because direct delivery of RNP is an efficient approach for CRISPR genome editing to lower off‐target effects and immunogenicity. The commonly used liposome‐based methods for RNP delivery have high transfection efficiency,^[11]^ but it is difficult to control the biodistribution and intracellular release of the RNP. The cytotoxicity of transfection reagents is also a concern. Gold nanoparticles (AuNPs) have been used for RNP delivery,^[12]^ but these methods usually involved coating of polymers or other ligands, which require complicated preparation processes. In addition, the proteins are randomly dispersed with different orientations, and therefore, it is difficult to maintain uniformity of the payload amount and the size of nanocarriers. Therefore, reliable and biocompatible delivery methods are still needed to ensure broad applications of protein coronae.

Construction of a BMPC comprises two main steps (Scheme 1 a): conjugation of affinity ligands onto the NPs of interest, and assembly of payload proteins onto the ligand‐conjugated NPs through affinity binding. The first step forms a ligand inner layer onto the NPs, and the second step generates a protein outer layer (Scheme 1). Various affinity ligands, such as antibodies, functional nucleic acids, and small molecules, can be used according to the payload proteins to be delivered.[^4^, ^13^] The ligand inner layer, containing dozen to hundreds of ligand molecules, plays two roles: providing affinity binding for the subsequent assembly of the payload proteins, and serving as a spacing layer between the NPs and the payload proteins. Specific binding of the payload proteins with the ligand inner layer assembles the payload proteins into a uniform outer monolayer. The density of the outer layer can be rationally controlled by affinity binding with appropriate amounts of the payload proteins, which provides the flexibility of modulating the interaction between the BMPC and the target cells.

We applied the BMPC technique to the delivery of RNP of the CRISPR‐Cas9 system, using AuNPs and magnetic nanoparticles (MNPs) as the scaffolds. We designed a DNA linker that hybridizes with the targeting region of the sgRNA and a short thiol‐labeled oligonucleotide (SH‐DNA), forming a ternary complex (Figure S1). To prepare BMPC, we first conjugated the ternary complex of oligonucleotides to AuNPs through gold‐thiol bonds,^[14]^ forming a ligand inner layer on AuNPs. sgRNA molecules in the inner layer served as affinity ligands for binding with the Cas9 protein. We assembled Cas9 over the inner layer by incubating the functionalized AuNPs with desired amounts of Cas9. The binding of sgRNA with Cas9 formed an oriented outer monolayer of RNP over the inner layer.

We designed the DNA linker to hybridize with the targeting region of the sgRNA, silencing the enzymatic activity of the RNP without affecting the binding affinity of sgRNA to Cas9. To achieve controllable release of the RNP from the BMPC, we introduced two photocleavable sites within the domain of the DNA linker hybridizing to the sgRNA. Therefore, spatio‐temporally controlled release of RNP can be achieved through the use of UV illumination to break the photo‐cleavable linker (PC linker).^[15]^

To examine the success of constructing BMPC with desired structures, we prepared two binding‐mediated RNP coronae by incubating Cas9 with sgRNA‐functionalized AuNPs (sgRNA‐AuNPs) at 10:1 and 25:1 molar ratios. We named them RNP corona (10:1) and RNP corona (25:1). We characterized them using transmission electron microscopy (TEM), dynamic light scattering (DLS), and ultraviolet‐visible (UV/Vis) absorption spectra. TEM images of the sgRNA‐AuNPs showed an additional faint shell, approximately 11.5 nm thick, around the AuNPs, suggesting the formation of the ligand inner layer (Figure 1 a). The shell increased from 11.4 nm to 24.2 nm and became denser for the RNP corona (10:1), due to the assembly of Cas9 onto the ligand inner layer and formation of the RNP outer layer. With increasing molar ratios of Cas9 to sgRNA‐AuNP from 10:1 to 25:1, the shell became both thicker (34.9 nm) and denser, suggesting that the density of the RNP layer and the size of the BMPC can be modulated by controlling the ratio of Cas9 to sgRNA‐AuNP. Additionally, the shape and density of the shells were similar among the specific RNP coronae, suggesting uniform RNP outer layers. The DLS results showed expected increases in the hydrodynamic sizes from AuNPs to sgRNA‐AuNPs and the binding‐mediated RNP corona (Figure 1 b). The larger hydrodynamic size of the fully assembled RNP corona is caused by the formation of the hydration shell and the binding of excess counterions.^[16]^ UV/Vis absorption spectra showed that the sgRNA‐AuNPs had a 2 nm redshift in optical absorption compared to that of the naked AuNPs (523 nm vs. 521 nm) due to changes in local refractive index. The assembly of RNP led to further redshifts of 4 nm (525 nm, RNP 10:1) and 7 nm (528 nm, RNP 25:1) (Figure 1 c). These consistent TEM, DLS, and UV/Vis results indicate successful formation of the BMPC.

**Figure 1:**
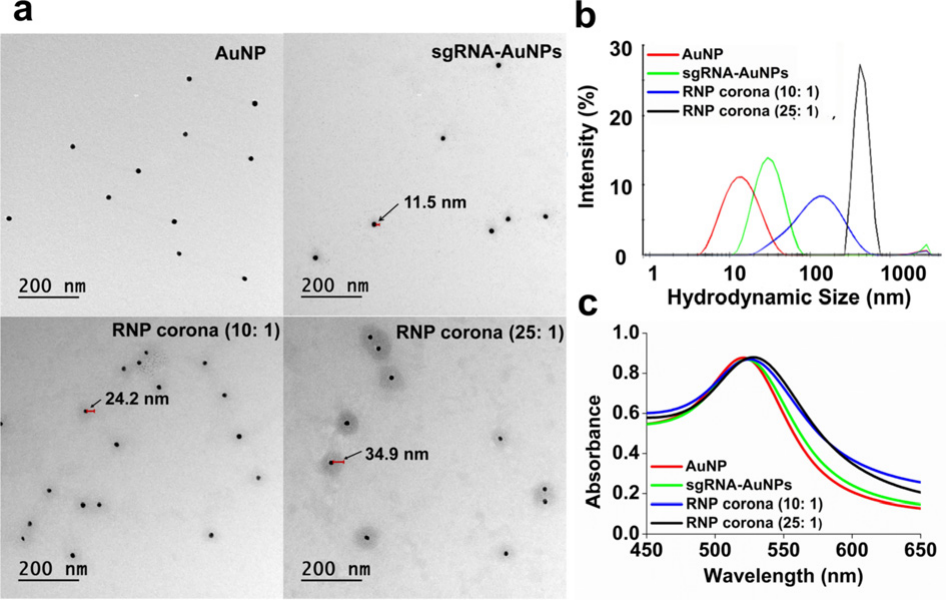
Characterization of AuNPs, sgRNA‐AuNPs, and the binding‐mediated RNP coronae (10:1) and (25:1). a) TEM images. Scale bar=200 nm. b) DLS measurement of hydrodynamic size distribution. c) UV/Vis absorption spectra.

To confirm that all added Cas9 molecules were fully assembled in the form of binding‐mediated RNP corona, we centrifuged the mixture and separately analyzed the supernatant and the precipitate (Figure S2). The gel electrophoresis results show that there was no leakage of Cas9 into the solution (Lanes A–C) and the Cas9 was completely assembled to form the corona and only detected in the AuNP precipitates (Lanes F–G).

To investigate the assembly efficiency and photoreactivity of the PC linker, we annealed the sgRNA, PC linker and SH‐DNA in an equal stoichiometric ratio. The appearance of one distinct band of expected mobility indicates successful assembly of the three oligonucleotide strands (Figure S3a). With irradiation (365 nm, 30 min), the sgRNA was completely dissociated from the assembly (Figure S3b), suggesting the photoreactivity of the PC linker.

We evaluated the light‐activated release and control of the RNP by monitoring the enzymatic cleavage of a 702 bp dsDNA fragment of the EGFP gene (enhanced green fluorescence protein). Our results (Figure S4, S5, and S6) confirmed the light‐activated release of RNP and the controllable activation of the Cas9‐sgRNA activity. The released RNP retained its nuclease activity to cleave the target DNA, with a comparable nuclease activity to that of the free RNP (Figures S4 and S6).

To test whether cellular uptake of the binding‐mediated RNP corona can be rationally modulated through control of density of the RNP outer layer, we prepared a set of the RNP coronae using different Cas9 to sgRNA‐AuNP ratios, 1:1, 3:1, 5:1, 10:1, 15:1, 20:1, and 25:1, and determined their uptake in Hela cells. We observed that the cellular uptake of the RNP coronae increased with increasing Cas9 to sgRNA‐AuNP ratio (Figure 2 a). Because a higher Cas9 to sgRNA‐AuNP ratio introduces more Cas9 molecules into the outer layer of the corona, the density increase of the RNP outer layer enhances the cellular uptake of the RNP coronae. After a 4‐h incubation of Hela cells with the RNP corona, the intracellular concentration of the RNP corona (25:1) was 250 nM, which was 12.5‐fold higher than that of sgRNA‐AuNPs and 25‐fold higher than the concertation of RNP corona in the incubation medium. Extending the incubation time to 8 h further improved intracellular concentration of the RNP corona (25:1) to 400 nM, equivalent to a 40‐fold enrichment over that in the culture medium (Figure S7). We also tracked the FAM‐labeled RNP corona using confocal microscopy images (Figure S8a) of Hela cells after incubation with the FAM‐labeled sgRNA‐Cas9, sgRNA‐AuNPs, or RNP corona for 4 h. Hela cells incubated with the RNP corona had significantly higher fluorescence signals, approximately 6‐fold higher than the cells incubated with sgRNA‐AuNPs (Figure S9). The cells incubated with the sgRNA‐Cas9 RNP had minimal fluorescence, indicating little uptake of the free RNP. Results from flow cytometry analysis were consistent (Figure S8b). After incubation with the RNP corona, 60.0 % of the cells were fluorescent, as compared to 39.5 % and 27.8 % after the cells were incubated with sgRNA‐AuNPs and the sgRNA‐Cas9 RNP, respectively. These results show that the RNP corona afforded efficient cellular uptake. More importantly, cellular uptake of the RNP corona were controllable by simply adjusting the density of the RNP on the outer layer of the BMPC.

**Figure 2:**
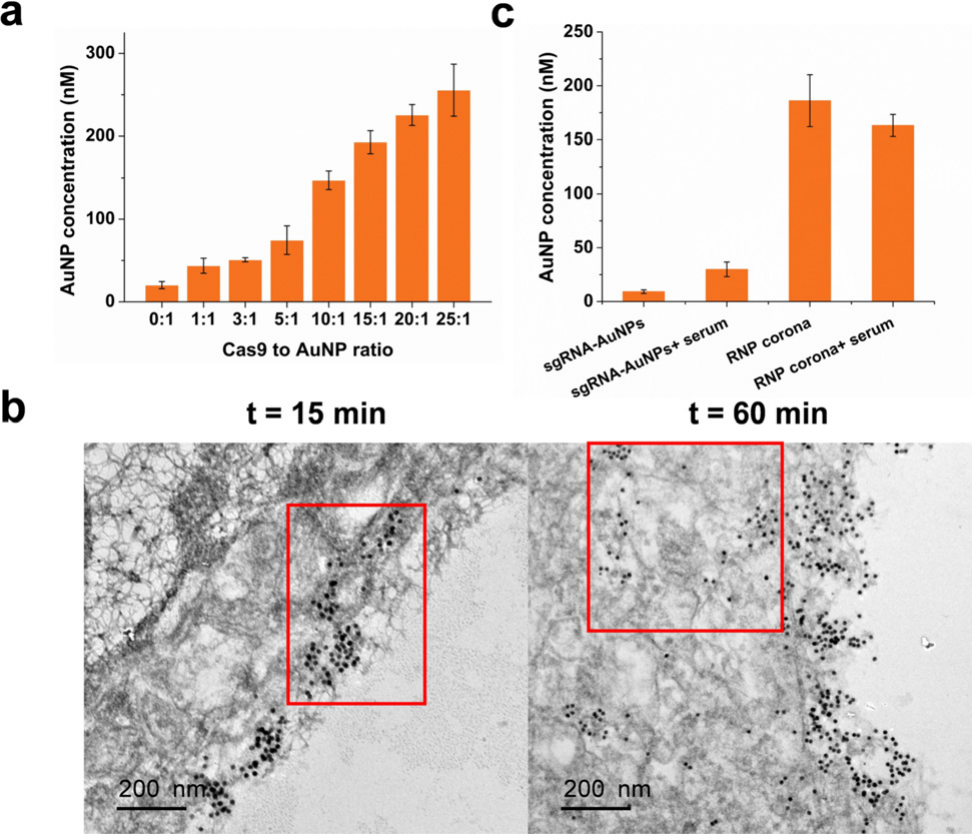
Cellular uptake of the binding‐mediated ribonucleoprotein corona. a) Quantification of cell uptake by measuring intracellular AuNPs using ICP‐MS. Cells were incubated for 4 h with different RNP coronae (10 nM) of increasing Cas9 density. b) TEM characterization of cell internalization of the RNP corona (10:1) after incubation (10 nM) with Hela cells for 15 min and 60 min. c) Cellular uptake of the RNP corona and sgRNA‐AuNPs in the presence and absence of 10 % FBS.

We reasoned that the efficient cellular uptake of the RNP corona was because of the enhanced interaction between the RNP corona outer layer and the cell membrane. We tested this hypothesis by tracking the cellular uptake of the RNP corona using TEM. TEM images show that the RNP coronae were localized on the membrane of Hela cells after 15 min of incubation (Figure 2 b). With prolonged incubation for 60 min, both cellular uptake and membrane localization of the RNP coronae were observed. These results suggest that strong interaction of the RNP coronae with the cell membrane might have mediated and promoted the cellular uptake of the RNP coronae. To further examine whether this enhanced cellular uptake is specifically attributed to the RNP outer layer, we prepared a non‐specific protein corona by incubating sgRNA‐AuNPs with 10 % fetal bovine serum (FBS) and compared it with the RNP corona for cellular uptake. Notably, the RNP corona had a ≈5‐fold higher uptake efficiency than this non‐specific corona formed with serum proteins (Figure 2 c). In addition, the presence of serum did not affect the uptake of the RNP corona, suggesting that the RNP corona remained stable during cellular uptake. The ≈5‐fold higher uptake efficiency of the RNP corona than the non‐specific corona formed with serum proteins (Figure 2 c) is not due to differences in protein density on the nanoparticles because the amounts of proteins per nanoparticle were similar (Figure S10).

To demonstrate that the enhanced cellular uptake of RNP corona is not due to the AuNP core, we used magnetic nanoparticles (MNPs) as the scaffold to construct another binding‐mediated RNP corona and determined its cellular uptake. Similarly, enhanced cellular uptake was observed with increasing Cas9 to MNP ratio (Figure S11). Therefore, efficient cellular uptake of the RNP corona is attributed to the RNP outer layer that enhanced the interaction between the RNP corona and the cell membrane.

We then investigated a possible mechanism of cellular uptake of the binding‐mediated RNP corona. Treatment with sodium azide (NaN_3_), a chemical reagent to disrupt the ATP production in cells and inhibit endocytosis,^[17]^ significantly decreased cellular uptake of the RNP corona (Figure S12), suggesting that the uptake of the RNP corona involves energy‐dependent endocytosis. To elucidate endocytosis pathway(s) involved in cellular uptake of the RNP corona, we tested five endocytosis inhibitors, including a clathrin‐mediated endocytosis inhibitor (chlorpromazine), two caveolin‐mediated endocytosis inhibitors (genistein and methyl‐β‐cyclodextrin), a phagocytosis inhibitor (cytochalasin D), and a receptor‐mediated endocytosis inhibitor (wortmannin).^[18]^ Except methyl‐β‐cyclodextrin, all the other inhibitors led to significant decreases in cellular uptake of RNP corona (Figure S13). These results suggest that multiple endocytosis pathways are involved in the cellular uptake of RNP corona.

To observe this endocytosis process, we labeled RNP corona with the fluorescent dye cyanine 5 (Cy5) and tracked its intracellular localization using confocal microscopy. The RNP coronae were mostly co‐localized with endosomes/lysosomes within 1 h of incubation, and then escaped from endosomes/lysosomes after incubation for 5 h (Figure 3 and S14, Figure S15). The Pearson correlation coefficient (Figure S14C) was 0.96±0.02, indicating overlap between the RNP corona and endosomes/lysosomes within 1 h of incubation. After incubation for 5 h, the Pearson correlation coefficient was 0.49±0.12, suggesting escape of the RNP from endosome. TEM images taken at 15 min, 1 h, and 5 h after incubation of cells with the RNP corona also show intracellular localization of RNP corona (Figure S15). Together, these results indicate that the endocytosis pathway contributed to cellular uptake of the RNP corona.

**Figure 3:**
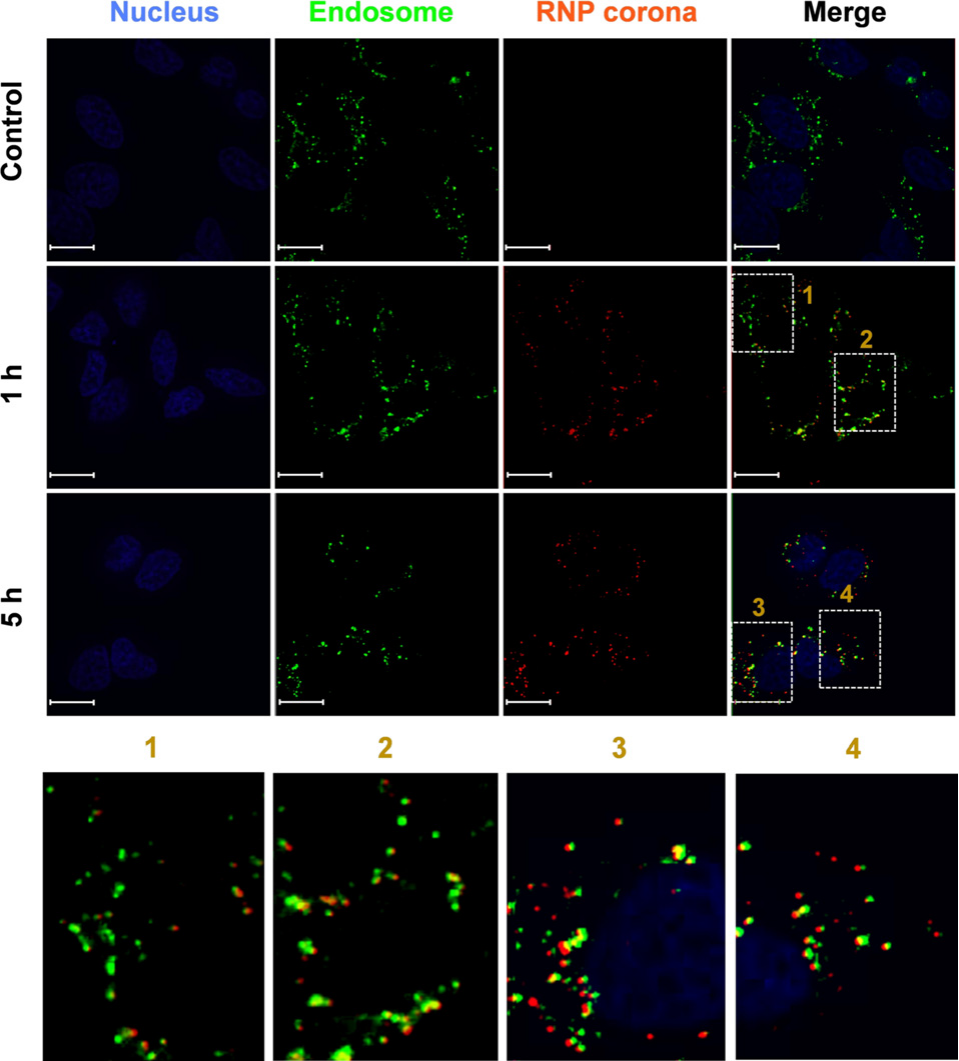
Endocytosis of the binding‐mediated RNP corona. See Figure S12 for experimental details. The endosomes/lysosomes were stained with LysoTracker Yellow HCK‐123 (50 nM, green) and the nuclei were stained with DAPI (4,6‐diamidino‐2‐phenylindole, blue). The RNP corona was red, due to the Cy5 labeling. Scale bar=15 μm.

To achieve genome editing in living cells using the BMPC technique, we first tested the stability of the RNP corona. The RNP corona exhibited an enhanced stability in both 10 % FBS and RNase A solution. In FBS solution, the free RNA reporter was quickly digested within 30 min, whereas only 20 % sgRNA was degraded from the RNP corona over one hour (Figure S16). The stability of the RNP corona in the RNase A solution was also improved compared to the stability of a RNA reporter, and the stability of the RNP corona increased with increasing Cas9 to sgRNA‐AuNP ratios (Figure S17). These results suggest that the corona protected sgRNA from degradation, a benefit for controlled gene editing over an extended period of time. We also examined the stability of RNP corona in the presence of 1–10 mM glutathione (GSH), representing the higher end of intracellular GSH concentrations. Our results show that RNP corona remained stable in the presence of 1–10 mM GSH (Figure S18).

Encouraged by the responsive release property and enhanced uptake efficiency of the RNP corona, we evaluated the gene editing potential of the activated RNP in Hela‐GFP cells. Successful editing of the EGFP transgene resulted in a loss of fluorescence, which was monitored using confocal microscopy and flow cytometry (Figure 4). We incubated the Hela‐GFP cells with the binding‐mediated RNP corona and measured fluorescence intensity three days after incubation. Without UV activation, the cells showed high fluorescence, which was as expected because the RNP was inactive. After UV irradiation, fluorescence of the cells decreased significantly, a result of successful delivery of the RNP corona into the cells, light activation of the RNP, and CRISPR knockdown of the GFP gene. Similar results were also observed from flow cytometry analysis: the number of fluorescent cells decreased from 41.8 % to 2.0 % after the cells were incubated with the RNP corona and UV irradiated (Figure 4 b). UV irradiation itself did not affect the GFP expression and fluorescence of the control cells (Figure S19). These results indicate that the genome editing activity of the RNP corona system was controllable using UV‐mediated activation. Moreover, the Hela cells incubated with the binding‐mediated RNP corona and irradiated with UV remained an overall 95 % viability (Figure S20), indicating low cytotoxicity of the light‐activated BMPC system.

**Figure 4:**
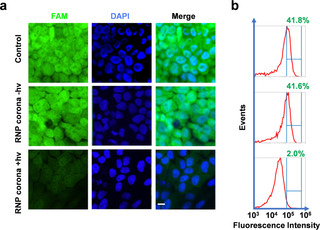
Editing of the EGFP gene in Hela‐GFP cells using the RNP corona system. a) Confocal microscopy images of Hela‐GFP cells. Cells were treated with RNP corona (ratio of 10:1, 10 nM) for 8 h, and irradiated at 3.0 mW cm^−2^ or kept in the dark for 15 min. The loss of fluorescence was measured five days after treatment. Scale bar=10 μm. b) Flow cytometry histograms of the Hela‐GFP cells corresponding to treatment in (a).

We also used the T7 endonuclease I (T7E1) mismatch cleavage assay to determine the editing efficiency of two genes in two cell lines (Figure 5), which supported the results of fluorescence measurements (Figure 4). Successful gene editing was observed in the Hela‐GFP cells after the light‐activated editing of the EGFP transgene (indel frequency 8.4 %) and the EMX1 gene (indel frequency 12.8 %) (Figure 5 a). No editing was observed in the control cells or the cells incubated with the RNP corona but not UV‐irradiated.

**Figure 5:**
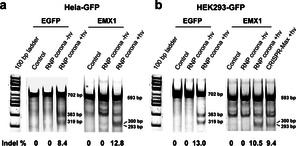
Editing efficiency of the EGFP gene and EMX1 gene in two human cell lines. The insertions and deletions (indel) frequency of both EGFP transgene (702 bp; cleaved to 383 bp and 319 bp) and human EMX1 gene (593 bp; cleaved to 300 bp and 293 bp) were measured in a) Hela‐GFP cells, b) HEK293‐GFP cells. The indel frequency was calculated by dividing the intensity of the fragment bands by that of the PCR input band. The indel frequency of the EMX1 gene using the RNP corona was compared with those using lipid‐mediated delivery method (CRISPR‐Max) in HEK293‐GFP cells.

We extended application of the RNP corona system to gene editing in the HEK293 cells (Figure 5 b), demonstrating the general applicability of the approach. Consistent indel frequencies of 12.8 % and 10.5 % were achieved for the EGFP and EMX1 genes, respectively. These editing efficiencies are comparable to or better than those achieved using the standard Lipofectamine transfection reagents (indel 9.4 %).

In summary, we have developed a binding‐mediated protein corona (BMPC) system for efficient delivery of functional CRISPR machinery and light‐activated genome editing. The RNP corona, which was formed with AuNPs, oligonucleotide ligand, and the sgRNA‐Cas9 ribonucleoprotein, protected the sgRNA from degradation and enhanced the delivery of the RNP into the cells. The RNP corona system exhibited low cytotoxicity to the Hela cells and maintained the nuclease activity of the RNP for gene editing. Incorporation of photocleavable linkers into the system enabled controlled activation of the sgRNA‐Cas9 RNP, resulting in photo‐responsive gene editing in human cells. In addition, because the Cas9 protein is not directly adsorbed onto the NPs, there is flexibility for encapsulating functional molecules, such as drugs and imaging probes, for therapeutic and biosensing applications. The BMPC strategy in combination with photo‐activation improves the delivery of RNP, offers optical control, and has potential for decreasing off‐target effects of the CRISPR‐Cas9 technology.

The BMPC technique has three appealing features: tunable outer layer density and property, minimized irreversible interaction of the payload proteins with the core of NPs, and reduced leakage of the payload proteins. These features make BMPC distinct from other techniques using NPs as carriers for delivering the RNP. Others used nonspecific interactions to load RNP onto AuNPs and/or included coating of an additional polymer layer, and the resulting RNP carriers could not afford uniform assembly of oriented Cas9 or tunable cellular uptake or controllable release. BMPC is advantageous for efficient and controllable delivery of the CRISPR‐Cas9 RNP system and is promising for delivery of nucleic acids and proteins with a high efficiency, stability, and activity.

## Conflict of interest

The authors declare no conflict of interest.

## Supporting information

As a service to our authors and readers, this journal provides supporting information supplied by the authors. Such materials are peer reviewed and may be re‐organized for online delivery, but are not copy‐edited or typeset. Technical support issues arising from supporting information (other than missing files) should be addressed to the authors.

Supplementary
