# Supplementary material for: Binding‐Mediated Formation of Ribonucleoprotein Corona for Efficient Delivery and Control of CRISPR/Cas9
Source: Angew Chem Int Ed Engl. 2021 Mar 18;60(20):11104–9. doi: 10.1002/anie.202014162 (PMC8252003; doi:10.1002/anie.202014162)
Supplement: Supplementary file 1 — Supplementary [file ANIE-60-11104-s001.pdf]

Angewandte Chemie
Eine Zeitschrift der Gesellschaft Deutscher Chemiker

Supporting Information

Binding-Mediated Formation of Ribonucleoprotein Corona for Efficient Delivery and Control of CRISPR/Cas9

Jinjun Wu[+], Hanyong Peng[+], Xiufen Lu, Maode Lai, Hongquan Zhang, and X. Chris Le**

anie_202014162_sm_miscellaneous_information.pdf

Supporting Information

Methods

Materials. Gold nanoparticles (15 nm) were purchased from Ted Pella (CA, USA). EnGen® Spy Cas9 NLS, HiScribe™ T7 high yield RNA synthesis kit, T7 endonuclease I, and Q5 high-fidelity 2× master mix were purchased from New England Biolabs Inc (MA, USA). Lipofectamine CRISPRMAX, TRIzol reagents, and LIVE/DEAD viability/cytotoxicity kit were purchased from Thermo Fisher Scientific (NH, USA). QIAquick PCR purification kit was obtained from QIAGEN (Germany). Tris-(2-carboxyethyl) phosphine hydrochloride (TCEP), β-mercaptoethanol, and sodium azide (NaN_3) were obtained from Sigma-Aldrich (MO, USA). N-Hydroxysuccinimide (NHS)-activated magnetic particles (MP) were obtained from Creative Diagnostics (NY, USA). Trypsin, penicillin streptomycin, phosphate-buffered saline (PBS), fetal bovine serum (FBS), Dulbecco's Modified Eagles Medium (DMEM), and Opti-MEM® I reduced-serum medium were purchased from Gibco (USA). 4′,6-diamidino-2-phenylindole (DAPI) was obtained from Invitrogen (CA, USA). All DNA sequences (Tables S1-4) were synthesized, labeled, and purified by Integrated DNA Technologies (CA, USA). FAM (carboxyfluorescein) and Cy5 were used in this study.

Synthesis of sgRNA. The double-stranded (ds) DNA template (121 bp) of single guide RNA (sgRNA) was generated using two long primers with 20-nt complementary sequences: (1) a 61-nt forward primer containing the T7 promoter (TAATACGACTCACTATA) and the 20-nt spacer sequence; (2) an 80-nt reverse primer encoding the 3′-end sequence of sgRNA (Table S3). Q5 High-Fidelity 2× Master Mix was used for synthesis of dsDNA template. The reaction was conducted in a thermocycler with the following cycling conditions: 95 °C for 2 min, 35 cycles of 95 °C for 20 s, 60 °C for 30 s, and 72 °C for 15 s, with a final extension period at 72 °C for 10 min. The templates were purified using QIAquick PCR purification kit and quantified using NanoVue Plus spectrophotometer (GE Healthcare). 500 ng of the purified template was added to the T7 *in vitro* transcription reaction and incubated at 37 °C for 16 h according to the manufacturer's protocol (https://international.neb.com/products/e2040-hiscribe-t7-high-yield-rna-synthesis-kit#Protocols,%20Manuals%20&%20Usage). The sgRNA was then purified using TRIzol® reagents and stored at -80 °C before use.

***In vitro* Cas9 nuclease cleavage assay.** A 702-bp dsDNA target was produced using PCR with the EGFP gene as the template (primer sequences in Table S3). This target was used to test the photo-responsive activity of the designed CRISPR Cas9 system. A mixture of 0.5 µL of 10 µM sgRNA, 10 µM photocleavable DNA linker (PC linker), and 10 µM thiol-labeled oligonucleotide (SH-DNA (Sequences in Table S2) was prepared with 1 µL of 10×NEB buffer and 7.5 µL of H_2O. The mixture was then heated in a thermal cycler to 80 °C for 5 min and cooled down to 25 °C with a -0.1 °C/s ramp rate. This procedure was used to hybridize the PC linker with the sgRNA and SH-DNA,

forming a generate the ternary complex. The performance of this "blocked sgRNA" was tested using the following procedures. The ternary complex (2 µL of 500 nM "blocked sgRNA") was mixed with 2 µL of 500 nM Cas9 protein, 1 µL of 10×NEB buffer, 4 µL of H_2O, followed by irradiation using a UV lamp (λ= 365 nm, 3.0 mW/cm^2) for 30 min. The dsDNA target of the EGFP gene (1 µL of 50 nM) was then added to the reaction mixture, followed by incubation at 37 ^oC for 1 h. This reaction cleaved the dsDNA target. The reaction was stopped by heating mixture at 75 ^oC for 15 min, which denatured the Cas9 protein. The reaction mixture was analyzed using polyacrylamide gel electrophoresis (PAGE) on a 4% native gel and at 100 V for 45 min. The gel was stained with SYBR Gold for 15 min and imaged using ImageQuant LAS4000.

Preparation of the binding-mediated ribonucleoprotein (RNP) corona using gold nanoparticles (AuNPs) as the scaffold. The "blocked sgRNA" (i.e., the ternary complex of SH-DNA, PC linker, and sgRNA) was first conjugated onto AuNPs. Specifically, 10 µL of 10 mM TCEP was mixed with 100 µL of 10 µM of the "blocked sgRNA", followed by incubation at room temperature for 1 h to reduce the disulfide bond in SH-DNA. The mixture was then added to 1 mL of 2 nM citrate-functionalized AuNPs solution containing 0.4 % Tween-80. The molar ratio of the "blocked sgRNA" to the AuNPs was 200: 1. After incubation for 30 min, 5 M sodium chloride was added to make a final concentration of 2 M NaCl. The solution was then incubated for 3 h with sonication for 30 s every half an hour. The AuNPs solution was then centrifuged (21,000g, 30 min) and washed for three times using a washing buffer (0.05% Tween-20 in 10 mM Tris-HCl, pH 7.4) to remove any excess "blocked sgRNA" ternary complex. The AuNPs were resuspended in 10 mM Tris-HCl buffer (pH 7.4). The Cas9 protein was then incubated with this functionalized AuNPs (sgRNA-AuNPs) at different measured ratios, and the incubation was at room temperature for 15 min. The interaction of the sgRNA with the Cas9 protein resulted in the formation of the ribonucleoprotein (RNP) corona.

Preparation of the binding-mediated ribonucleoprotein (RNP) corona using magnetic nanoparticles (MNPs) as the scaffold. The amine-labeled DNA (NH_2-DNA) was used to replace SH-DNA in the preparation of the ternary complex, by annealing the PC linker with the sgRNA and the NH_2-DNA, forming the "blocked sgRNA" (sequences in Table S2). The NHS-activated magnetic nanoparticles (50 nm, 1 pmol) were washed with 1 mL of 1× PBS buffer for three times. Hydrochloric acid (1 mL of 1 mM) was then added to the MNPs, followed by gentle vortex for 15 s, to activate the NHS-group. The activated NHS-group on the MNPs facilitated the reaction between NHS esters and the amine group of NH_2-DNA. A 100-µL aliquot of 10 µM "blocked sgRNA" was added to the MNPs solution to conjugate the "blocked sgRNA" to the MNPs. After incubation on a rotator at room temperature for 2 h, the MNPs were treated with 3 M ethanolamine for 30 s to deactivate the NHS groups. The MNPs were washed and resuspended in 10 mM Tris-HCl buffer (pH 7.4). The Cas9 protein was then incubated with the functionalized MNPs (sgRNA-MNPs) at different ratios (100: 1 or 500: 1) at room temperature for 15 min.

Determination of the loading amount of the sgRNA onto the AuNPs (Figure S5). The 3'-end of the "blocked sgRNA" was hybridized with a FAM-labeled antisense DNA (FAM-26 on Table S2) before the ternary complex was conjugated onto the AuNPs. The fluorescence of FAM was quenched by AuNPs when the complex was attached to the AuNPs. When the complex was released from the AuNPs, the

fluorescence was no longer quenched. Fluorescence measurement provided information on the loading amount of the sgRNA on the AuNPs. A mixture of 10 µL of 35 mM 2-mercaptoethanol and 80 µL of 1× PBS was added to 10 µL of 2 nM functionalized sgRNA-AuNPs to release the sgRNA complex from the AuNPs. After incubation at room temperature overnight, the AuNPs aggregated and precipitated. The supernatant (95 µL) containing the released sgRNA complex was then transferred to a 96-well plate and the fluorescence was measured using a fluorescence plate reader (Beckman Coulter, DTX 800) with excitation at 485 nm and emission at 535 nm. Our results showed that on average 40 molecules of the ternary complex (the “blocked sgRNA”) were conjugated on each AuNP.

Determination of the loading efficiency of Cas9 on the sgRNA functionalized AuNPs (Figure S2). A 6-µL aliquot of the functionalized sgRNA-AuNPs (50 nM) was mixed with different volumes of 1 µM Cas9, achieving molar ratios of Cas9 to AuNPs being 5: 1, 10: 1 and 25: 1. To this solution was added 10 mM Tris-HCl buffer (pH 7.4) containing 1 mM $MgSO_4$ to a final volume of 15 µL. After incubation at room temperature for 15 min, the solution was centrifuged at 21,100 g for 30 min. The AuNPs precipitates and the supernatant were separately analyzed for the presence of Cas9. The supernatant and AuNPs precipitates were separately treated with a denaturing buffer (2% SDS, 5% β-mercaptoethanol, 10% glycerol, 0.001% bromophenol blue, and 62.5 mM Tris-HCl pH 6.8) and heated at 95^oC for 5 min. Each of the treated solutions was analyzed for the Cas9 protein using sodium dodecyl sulfate (SDS) polyacrylamide gel electrophoresis (PAGE).

Light-activated control and release of the RNP (Figures S3-S6). The PC linker, sgRNA, and SH-DNA were annealed in an equal stoichiometric concentration. The appearance of one distinct band of expected mobility indicates successful assembly of the three oligonucleotide strands (Figure S3a). With irradiation (365 nm, 30 min), the sgRNA was completely dissociated from the assembly (Figure S3b), suggesting the photoreactivity of the PC linker.

The photoactivation of the “blocked sgRNA” Cas9 RNP complex was tested by monitoring the *in vitro* nuclease cleavage of a 702-bp dsDNA fragment of the EGFP gene (enhanced green fluorescence protein, before and after light activation (Figure S4). Without UV illumination, the sgRNA was hybridized to the PC linker and not available to hybridize with the dsDNA target. Therefore, the RNP was not active and the 702-bp dsDNA target remained intact (Figure S4, lane 3). After UV illumination, the PC linker was cleaved and dissociated from the sgRNA. The sgRNA strand in the RNP became freely available to hybridize with the target DNA, resulting in the activation of the RNP. The nuclease function of the active RNP led to the observed 383 bp and 319 bp fragments of the 702 bp target (Figure S4, lane 4). The light-activated RNP had a comparable nuclease activity to that of the free RNP (Figure S4, lane 2). These results indicate photo-responsive availability of the sgRNA and controllable activation of the Cas9-sgRNA activity (Figure S4).

Figure S5 show the light-activated release of the sgRNA and RNP from AuNPs. The sgRNA complex was labeled at the 3'-end by hybridization with a FAM-labeled 26-nt oligonucleotide (FAM-26) (Figure S5a). Without UV irradiation, the fluorescence was low because AuNPs quenched the fluorescence of FAM on the surface of AuNPs. With UV illumination, the FAM-labeled sgRNA complex and the FAM-labeled RNP complex were released from the AuNPs, resulting in the recovery of fluorescence

(Figure S5b).

The light-activation of the RNP coronae was observed by monitoring the nuclease cleavage of the target DNA by the released RNP (Figure S6). Without UV illumination, the RNP corona was inactive because the sgRNA was hybridized to the PC linker and not available to interact with the dsDNA target. Therefore, the 702-bp dsDNA target remained intact (Figure S6, lane 2). After UV illumination, all three RNP coronae (5:1, 10:1, and 25:1) became active, and the target DNA was cleaved by the active RNP to the 383-bp and 319-bp fragments (Figure S6, lanes 3-5). These results indicate controllable activation of the RNP corona.

Stability of the binding-mediated RNP coronae. The stability of the RNP coronae in fetal bovine serum (Figure S16) and in RNase A (Figure S17) was compared with the stability of a fluorescent RNA reporter (RNaseAlert). The sgRNA in the RNP corona was labeled with a FAM-labeled complementary DNA (FAM-26), so that the fluorescence could be detected if the sgRNA was cleaved off from the AuNPs. The RNA reporter (RNaseAlert) was dually labeled with a pair of FAM and quencher on either end of the reporter sequence, so that the fluorescence of FAM could be detected when the RNA reporter was cleaved and the quencher separated from FAM. A 10-µL aliquot of 10 nM RNP corona (10: 1) or 10 µL of 40 nM RNA reporter (RNaseAlert) was mixed with 10 µL of 10 ×PBS, 10 µL of FBS, and 70 µL of H_2O. Each of the solutions was transferred to a 96-well plate and the fluorescence was measured every 10 min for 1 h at room temperature (Figure S16). The cleavage percentage was obtained by comparing the measured fluorescence with total fluorescence. The total fluorescence was determined after treating a parallel sample with 2-mercaptoethanol.

Similarly, a 10-µL solution of 10 nM RNP corona (10: 1) or 10 µL of 40 nM RNA reporter was mixed with 90 µL of 1×PBS containing 70 micro-units (µunits) of RNase A. Each of the treated solutions was transferred to a 96-well plate, and the fluorescence was measured every 30 s (Figure S17).

Cell culture. All cell lines used in this study, including Hela cells, Hela-GFP cells and HEK293-GFP cells, were maintained in DMEM supplemented with 10% FBS, 1% penicillin/streptomycin, and 1% non-essential amino acids. Cells were cultured in a humidified incubator at 37 ^oC in the presence of 5% CO_2. Puromycin (1 µg/mL) was added to Hela-GFP cells and blasticidin (10 µg/mL) was added to HEK293-GFP cells to inhibit the growth of non-GFP cells.

Quantification of cellular uptake of RNP coronae using ICP-MS (Figure S7). The cellular uptake of the RNP coronae and the functionalized AuNPs was quantified by measuring AuNPs in the cells using ICP-MS. Hela cells were seeded in a 24-well plate at a density of 1×10^5 cells per well one-day prior to treatment to ensure adhesion of cells onto the tissue-culture plate surface. Cells were washed with 1× PBS and then incubated with the binding-mediated RNP coronae or the sgRNA functionalized AuNPs (sgRNA-AuNPs) at different concentrations (0.5, 1, 2, 5, 10 nM) in Opti-MEM for different time periods (1, 2, 4, 6, 8 h). After cellular uptake, the cells were thoroughly washed with 1×PBS six times, then detached, and counted using a hemocytometer. The cells were then digested in 20% HNO_3 at 60 ^oC overnight. The concentration of Au was measured using an inductively coupled plasma mass spectrometer (Agilent Technologies, Japan). The concentration of Au was calibrated against a standard of digested AuNPs. The intracellular concentration of AuNPs was obtained by dividing

the measured amount of AuNPs by the number of cells and the average cell volume. The cell size of 20 μm in diameter was used to estimate the cell volume, and for simplicity the shape of cells was considered as a sphere.

For studies of the cellular uptake mechanism, the cells were pretreated with 50 nM NaN_3 for 1 h before incubation with the binding-mediated RNP coronae or the sgRNA functionalized AuNPs (sgRNA-AuNPs) (Figure S11). The subsequent procedures of cell treatment and ICP-MS analysis were the same as described above.

Fluorescence measurement of cellular uptake (Figure 3, Figure S8, Figure S14). The cellular uptake of the sgRNA-Cas9 RNP, the sgRNA functionalized AuNPs (sgRNA-AuNPs), and the binding-mediated RNP coronae was determined by monitoring the intracellular fluorescence of the FAM-labeled sgRNA using confocal microscopy and flow cytometry. Briefly, cells were seeded in an 8-well μ-slide at a density of 40,000 cells per well one-day prior to treatment. Next, the cells were incubated with the sgRNA-Cas9 RNP (100 nM), sgRNA-AuNPs (10 nM), or the RNP corona (10:1) (10 nM) for 4 h, and then washed with 1×PBS three times. DAPI (1.0 μg/mL) was used to stain the cell nuclei, and LysoTracker Yellow HCK-123 (50 nM) was used to stain the endosomes/lysosomes. Fluorescence images were captured using a confocal microscope (Quorum Technologies, ON).

GFP knockdown using the binding-mediated RNP corona (Figure 4). Hela-GFP cells were seeded in an 8-well μ-slide at a density of 40,000 cells per well and cultured overnight. The cells were washed and incubated with 100 μL of 10 nM RNP corona (10:1) in Opti-MEM for 8 h and then UV irradiated for 15 min. The medium was replaced with 200 μL of fresh DMEM and incubated for five days. The cells were analyzed using confocal fluorescence microscopy and flow cytometry.

T7E1 assay (Figure 5). Genome DNA was extracted from cells using a QuickExtract genomic DNA isolation kit (Lucigen). The target regions (EGFP and EMX1) of genome DNA were PCR amplified using Q5 High-Fidelity 2× Master Mix and subjected to T7E1 assay according to the manufacturer's instruction (EnGen® Mutation Detection Kit, NEB). Briefly, 100 ng of the extracted genome DNA was mixed with 12.5 μL of Q5 Hot Start High-Fidelity 2× Master Mix, 1.25 μL of 10 μM forward primer, 1.25 μL of 10 μM reverse primer, and 10 μL of H_2O. The following PCR cycling conditions were used: 98 ^oC for the initial 1 min, followed by 35 cycles of 98 °C for 5 s, 67 °C for 10 s and 72 °C for 20 s, and a final extension period of 2 min at 72 °C. A 5-μL aliquot of the PCR product was mixed with 2 μL of 10×NEB buffer and 12 μL of H_2O. The mixture was heated at 95 ^oC for 5 min, cooled down to 85 ^oC at a rate of -2^oC/sec and further to 25^oC at a rate of -0.1^oC/sec. The T7 endonuclease I enzyme (1 μL of 10 U/μL) was added to the annealed PCR product. After incubation at 37 ^oC for 30 min, 1 μL of Proteinase K was added to the solution. The solution was further incubated at 37 ^oC for 5 min to inactivate the T7 endonuclease I. A 10-μL aliquot of the reaction products was loaded to an 4% native PAGE. Gel electrophoresis was performed at 100 V for 45 min. The gel was stained with SYBR Gold for 15 min and visualized using ImageQuant LAS4000. The intensity and area of the bands were estimated using ImageJ. The insertions and deletions (indel) frequency was calculated by dividing the intensity of the fragment bands by that of the PCR input band.

Cell viability assay (**Figure S20**). Hela cells (40,000 cells) were either incubated with 10 nM RNP corona (10:1) or control for 4 h. The cells were either not treated or UV-

irradiated (λ= 365 nm, 3.0 mW/cm^2) for 30 min. The cells were washed, and incubated in the fresh DMEM medium for 24 h. The cells were detached using 0.05% trypsin-EDTA and stained with 10 μL of the reagent in Live/Dead viability/cytotoxicity kit (ThermoFisher) for 30 min. The fluorescence intensity of cells was then analyzed using flow cytometry.

Table S1: Sequences of the sgRNA target site. The CGG and GGG sequences in blue color are the PAM sequences.

**Genome target**	**Target site sequence (5'-3')**
EGFP	GGCATCGACTTCAAGGAGGACGG
EMX1	GAGTCCGAGCAGAAGAAGAAGGG

Table S2: Sequences used to form the ternary complex of PC linker, SH-DNA (or NH_2-DNA), and sgRNA. The complex is also referred to as the "blocked sgRNA" because the binding region of the sgRNA is hybridized to the PC linker and the sgRNA is "blocked" from interacting with its target DNA. FAM-26 and Cy5-26 are the sequences used to provide a fluorescence label, by hybridizing these fluorescent sequences to the 3'-end of the sgRNA (Figure S5a and Figure S14a).

**Name**	**Sequences (5'-3')**
SH-DNA	HS-TTGCAGAGAGACAGGTTACA
NH_2-DNA	H_2N-TTGCAGAGAGACAGGTTACA
PC linker	CTCCTCC*TTGAAGT*CGATGCCTGTAACCTG TCTCTCTGC
sgRNA-GFP	GGCAUCGACUUCAAGGAGGAGUUUUAGAGCUA GAAAUAGCAAGUUAAAAUAAGGCUAGUCCGUU AUCAACUUGAAAAAGUGGCACCGAGUCGGUGC UUUU
sgRNA-EMX1	GAGUCCGAGCAGAAGAAGAAGUUUUAGAGCUA GAAAUAGCAAGUUAAAAUAAGGCUAGUCCGUU AUCAACUUGAAAAAGUGGCACCGAGUCGGUGC UUUU
FAM-26	FAM-AAAGCACCGACTCGGTGCCACTTTTT
Cy5-26	Cy5-AAAGCACCGACTCGGTGCCACTTTTT

Table S3: T7 promoter containing sequences for the synthesis of sgRNA.

Primer name	Primer sequences (5'-3')
sgRNA-GFP Forward primer	TCTAATACGACTCACTATAGGGCATCGACTTCAAGGAGGAGTTTTAGAGCTAGAAATAGCA
sgRNA-EMX Forward primer	TCTAATACGACTCACTATAGGAGTCCGAGCAGAAGAAGAAGTTTTAGAGCTAGAAATAGCA
Reverse primer	AAAAGCACCGACTCGGTGCCACTTTTTCAAGTTGATAACGGACTAGCCTTATTTTAACTTGCTATTTCTAGCTCTAAAAC

Table S4: Primer sequences for PCR amplification of the target genes.

Primer name	Gene target	Primer sequences (5'-3')
FP-GFP	EGFP	GAGGAGCTGTTCACCGGG
RP-GFP	EGFP	CTTGTACAGCTCGTCCATGC
FP-EMX	EMX1	CTGTGTCCTCTTCCTGCCCT
RP-EMX	EMX1	CTCTCCGAGGAGAAGGCCAA

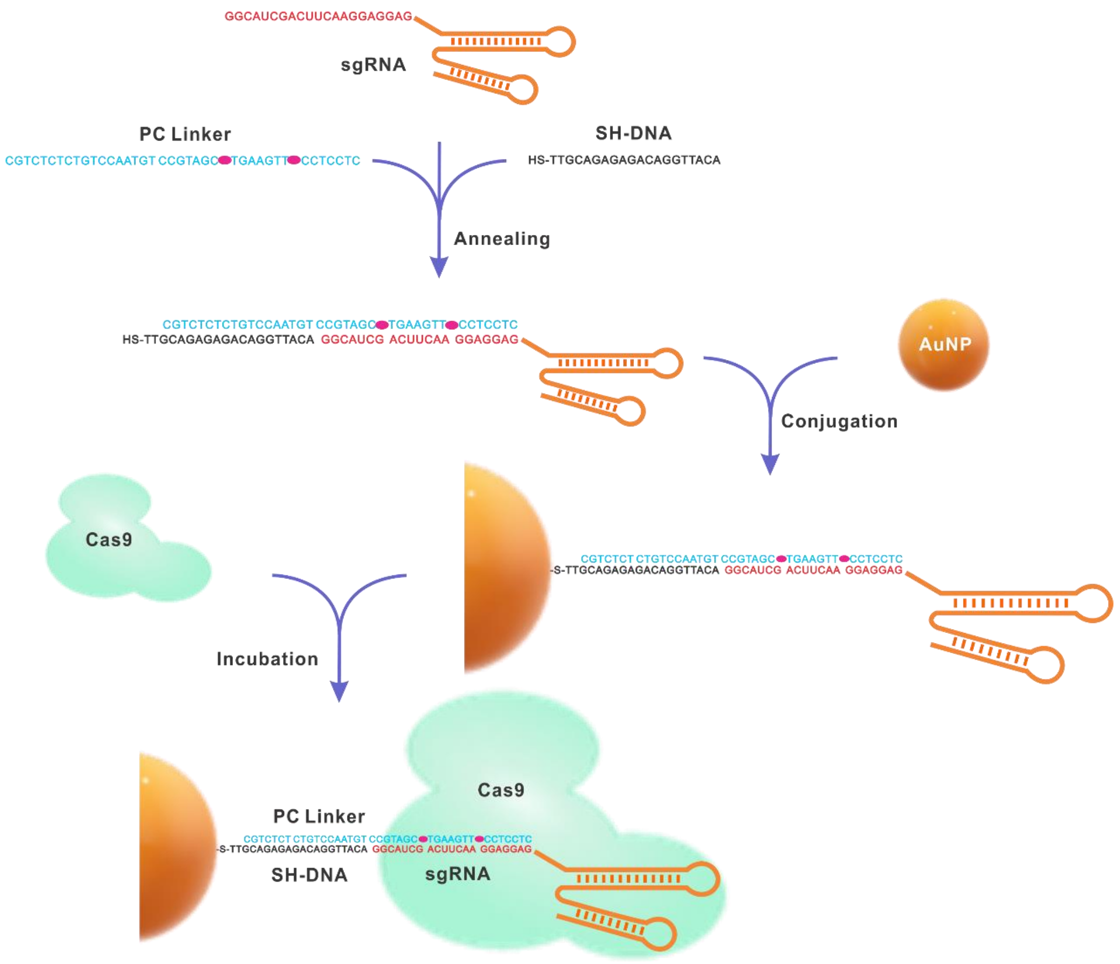

Figure S1. Design and sequences used in the light-activated ribonucleoprotein corona delivery system. A photocleavable DNA linker (PC linker, blue) consisting of two cleavage sites (purple) is complementary to the binding region of sgRNA (red) and a thiol-labeled DNA (SH-DNA, black). The sgRNA, the PC linker, and the SH-DNA are annealed, resulting in the hybridization of the PC linker with the sgRNA and the SH-DNA, forming a ternary complex. The ternary complex is conjugated to the gold nanoparticle (AuNP) using the reactive thiol-group in the SH-DNA. The ternary complex is also referred to as the "blocked sgRNA" because the binding region of the sgRNA is hybridized to the PC linker and the sgRNA is "blocked" from interacting with its target DNA. The AuNPs functionalized with the "blocked sgRNA" are incubated with the Cas9 protein. The interaction of Cas9 with the sgRNA results in the formation of sgRNA-Cas9 ribonucleoprotein (RNP) corona. This binding-mediated formation of RNP corona is controllable by incubating Cas9 and the sgRNA functionalized AuNPs at desirable ratios, giving rise to the RNP coronae (5:1), (10:1), and (25:1).

The PC linker contains two photo-cleavable sites. UV illumination photolyzes the PC linker and destabilizes the hybridization between the PC linker and the sgRNA, resulting in the release of the ribonucleoprotein (RNP) from the AuNPs. The binding region of the sgRNA in the released RNP is freely available to interact with its target DNA. Thus, the released RNP is active for CRISPR genome editing.

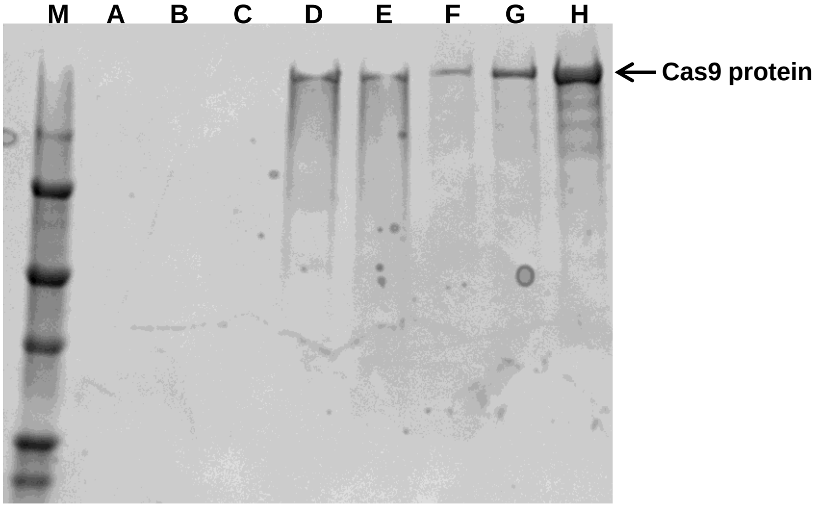

Figure S2. Analysis of Cas9 in the supernatant (A-C) and the AuNPs precipitates (F-H) indicating complete loading of Cas9 on the sgRNA functionalized AuNPs. Cas9 was mixed with the sgRNA functionalized AuNPs (sgRNA-AuNPs) at a Cas9 to the functionalized AuNP ratio of 5:1, 10:1, or 25:1. After incubation at room temperature for 15 min, the solution was centrifuged to precipitate AuNPs. The supernatant and the precipitates were separately heated at 95 ^{o}C for 5 min and separately analyzed for Cas9 using SDS-PAGE. Lane M: markers; lanes A-C: supernatants of three Cas9 to AuNP ratios (5:1, 10:1, and 25:1); lane D: Cas9 solution; lane E: supernatant of Cas9 solution after centrifugation; lanes F-H: Cas9 released from the AuNP precipitates of three RNP preparations with different Cas9 to the functionalized AuNP ratios being 5:1, 10:1, and 25:1. These results indicate that Cas9 was completely loaded on the sgRNA functionalized AuNPs.

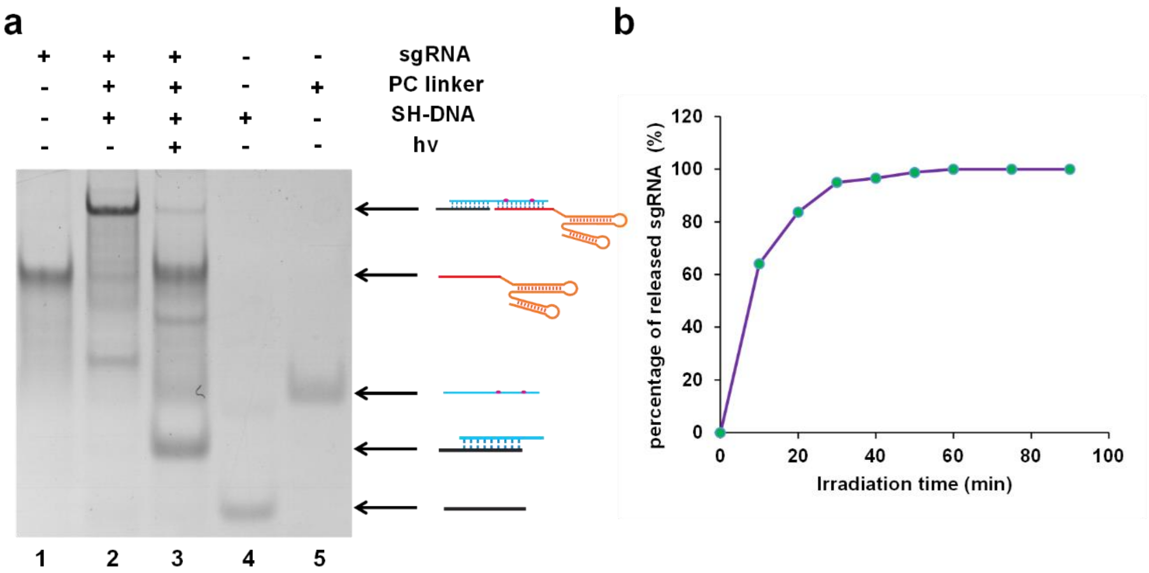

Figure S3. Formation and photo-release of the "blocked sgRNA". (a) Gel electrophoresis images showing the sgRNA, the PC linker, the SH-DNA, and their hybridization products. Lane 1: sgRNA; lane 2: "blocked sgRNA" (anneal sgRNA, SH-DNA and PC linker at a 1: 1: 1 ratio) without UV illumination; lane 3: "blocked sgRNA" after 30 min of UV illumination (365 nm, 3.0 mW/cm^2); lane 4: SH-DNA; lane 5: PC linker. Annealing was carried out by heating the mixture of the three oligonucleotides to 80 ^{o}C for 5 min and cooling down to 25 ^{o}C at a rate of -0.2^{o}C/sec. Gel electrophoresis was run on 12% native PAGE (100 V, 45 min) at room temperature. (b) The photo-release efficiency of sgRNA. The "blocked sgRNA" was irradiated under the UV light (365 nm, 3.0 mW/cm^2) for different time periods (10, 20, 30, 40, 50, 60, 70, 80, 90 min). The products were analyzed using a 2% native PAGE (100 V, 45 min). The intensity of the gel bands was estimated using the ImageJ software and used to estimate the percentage of released sgRNA.

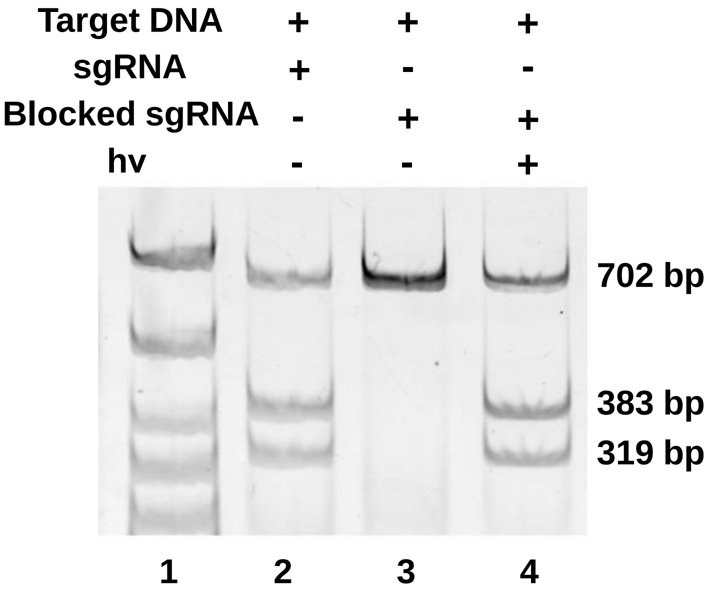

Figure S4. Gel electrophoresis of a 702-bp EGFP DNA target and its cleavage products (383 bp and 319 bp) generated by the sgRNA-Cas9 nuclease activity. The free sgRNA or the “blocked sgRNA” (2 µL, 500 nM) was mixed with 2 µL of 500 nM Cas9 and 5 µL of 2×NEB buffer. The mixture was either not UV-irradiated or UV irradiated (λ= 365 nm, 3.0 mW/cm^2) for 30 min (hv – or +). To each solution was added 1 µL of 50 nM EGFP DNA target (702 bp). After incubation at 37 ^{o}C for 1h to allow for the CRISPR nuclease reaction, the solution was heated to 75 ^{o}C for 15 min to denature the Cas9 protein. The reaction mixture was analyzed using polyacrylamide gel electrophoresis (PAGE) on a 4% native gel and at 100 V for 45 min. The gel was stained with SYBR Gold for 15 min and imaged using ImageQuant LAS4000. The far right lane show that the photo-activated RNP cleaved the EGFP DNA target (702 bp) into two fragments (383 bp and 319 bp).

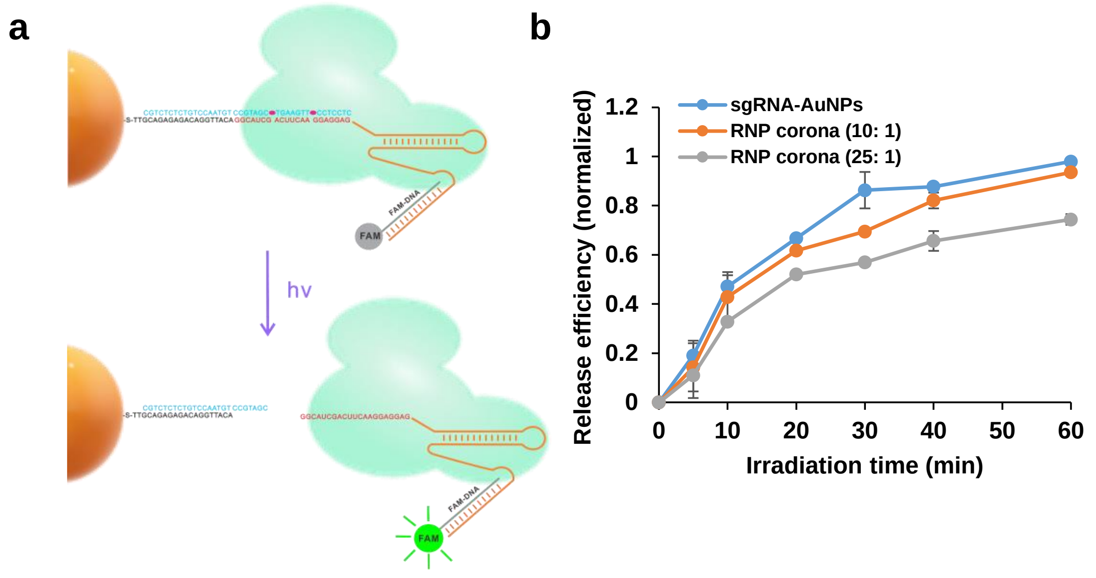

Figure S5. Fluorescent labeling of the sgRNA complex (a) and monitoring the release of sgRNA from the functionalized AuNPs or the binding-mediated RNP coronae in response to UV irradiation. (a) One domain at the 3'-end of sgRNA was hybridized with a FAM-labeled complementary DNA (FAM-26, sequence in Table S2). The fluorescent labeling of the sgRNA complex with FAM was for assessing the release of the sgRNA and for tracking the uptake of the RNP coronae. This construct was used to generate the results shown in Figure S8. (b). Release of the sgRNA complex from three constructs: the sgRNA functionalized AuNPs, the binding-mediated RNP corona (10:1), and the binding-mediated RNP corona (25:1).

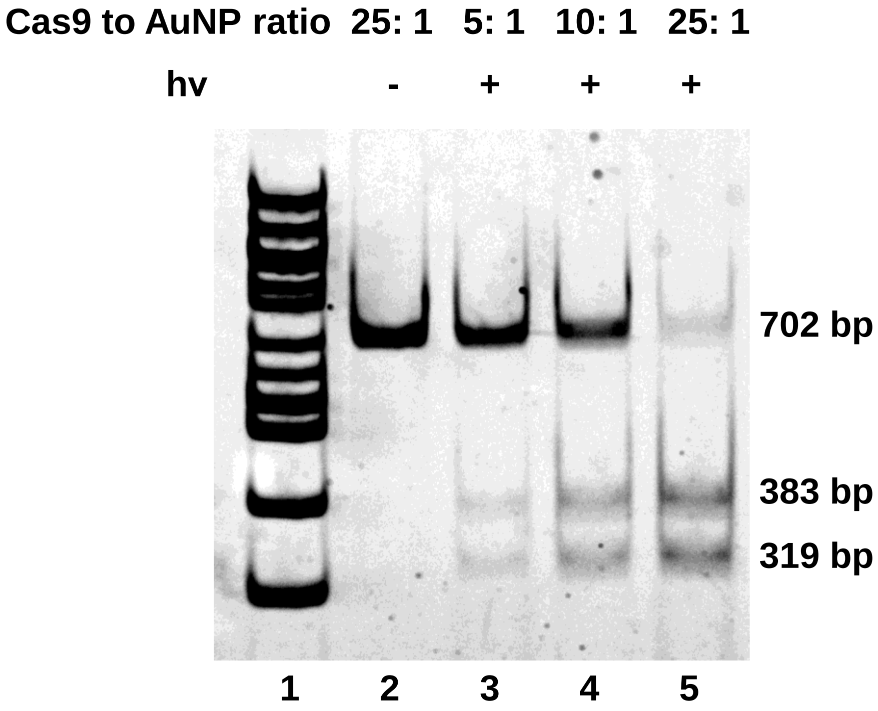

Figure S6. Gel electrophoresis of a 702-bp EGFP DNA target and its cleavage products (383 bp and 319 bp) generated by the RNP corona. Three binding-mediated RNP coronae were prepared using three molar ratios of the Cas9 to AuNPs, 5:1, 10:1, and 25:1. The RNP coronae were either UV irradiated (λ= 365 nm, 3.0 mW/cm^2) for 30 min or not irradiated (hv + or –). To each solution was added 1 µL of 50 nM EGFP DNA target (702 bp). After incubation at 37 °C for 1 h to allow for the CRISPR nuclease reaction, the solution was heated to 75 °C for 15 min to denature the Cas9 protein. The reaction mixture was analyzed using polyacrylamide gel electrophoresis (PAGE) on a 4% native gel and at 100 V for 45 min. The gel was stained with SYBR Gold for 15 min and imaged using ImageQuant LAS4000.

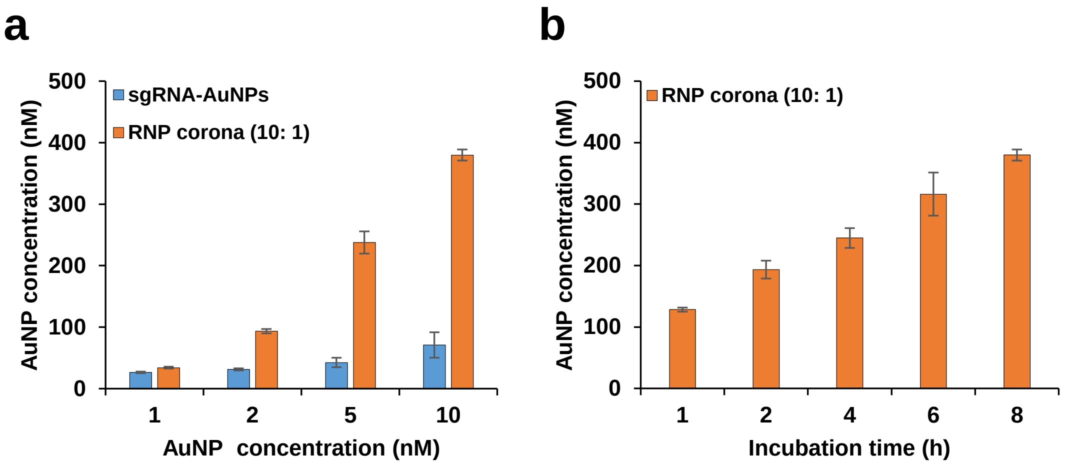

Figure S7. Uptake of the binding-mediated RNP corona by Hela cells as a function of the concentration or incubation time. (a) Comparison of cellular uptake of the sgRNA-functionalized AuNPs (sgRNA-AuNPs) and the RNP corona (10:1) of four concentrations (1, 2, 5, and 10 nM). The Hela cells were incubated with either the sgRNA-AuNPs or the RNP corona (10:1) for 8 h. (b) Cellular uptake of the binding-mediated RNP corona (10:1) over the incubation time from 1 to 8 h. The Hela cells were incubated with 10 nM of RNP corona (10:1). The intracellular concentrations of AuNPs were determined using inductively coupled plasma mass spectrometry (ICP-MS).

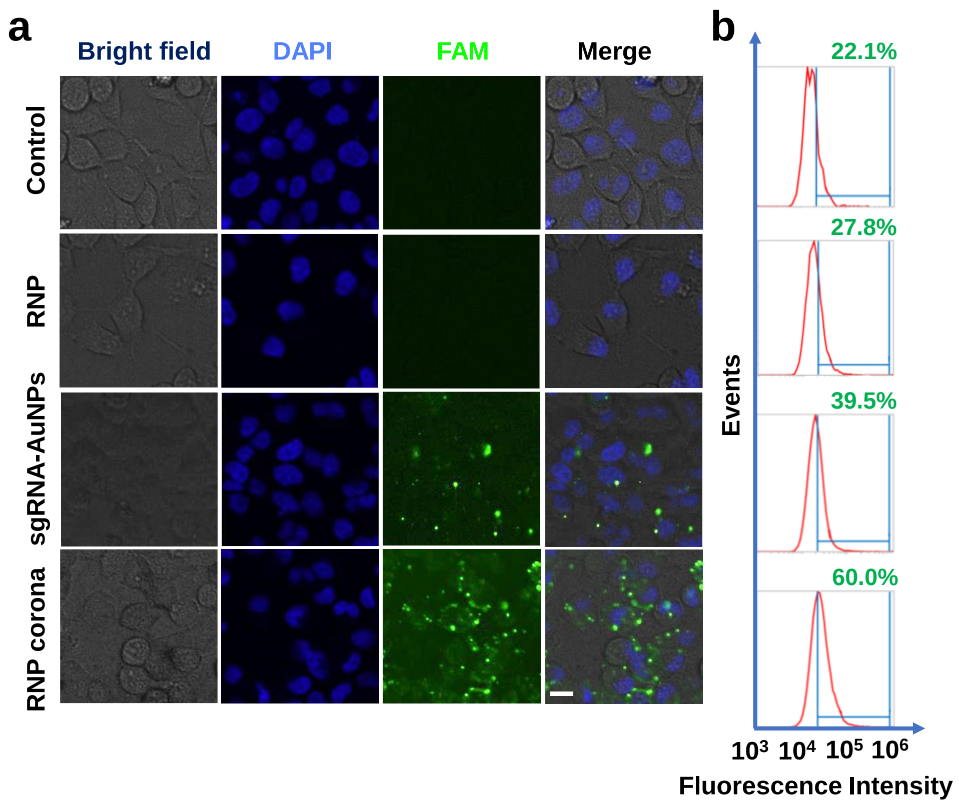

Figure S8. Fluorescence monitoring of cellular uptake of the sgRNA-Cas9 RNP, the sgRNA functionalized AuNPs (sgRNA-AuNPs), and the binding-mediated RNP corona. (a) Confocal microscopy images of Hela cells after treatment with the free RNP (100 nM), sgRNA-AuNPs (10 nM) or the RNP corona (10:1) (10 nM) for 4 h. The sgRNA was labeled with FAM as in Figure S5a. The nucleus was stained with 4′,6-diamidino-2-phenylindole (DAPI). The scale bar is 10 μm. (b) Flow cytometry histograms detecting cells with intracellular FAM-labeled molecules corresponding to treatments in (a).

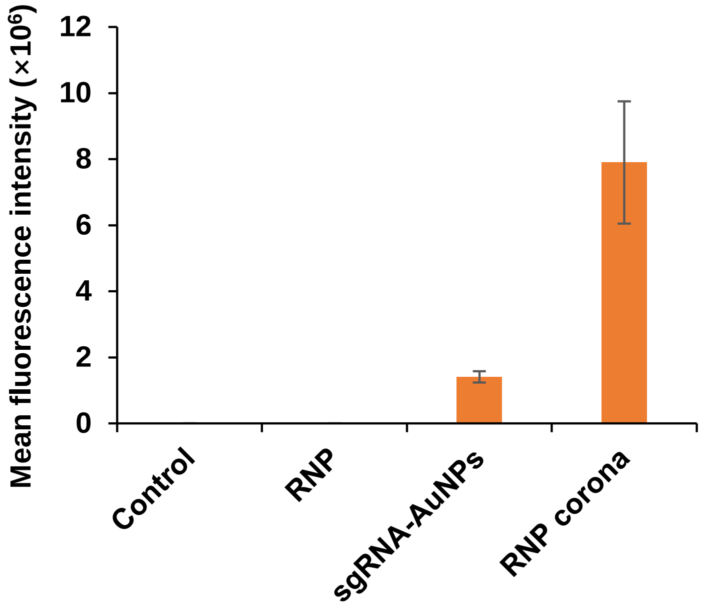

Figure S9. Fluorescence intensity of the control cells and the cells incubated with the sgRNA-Cas9 RNP, the sgRNA functionalized AuNPs (sgRNA-AuNPs), and the binding-mediated RNP corona. The fluorescence intensities of confocal images of Hela cells was measured using the ImageJ software. The cell treatment conditions were the same as described in Figure S8.

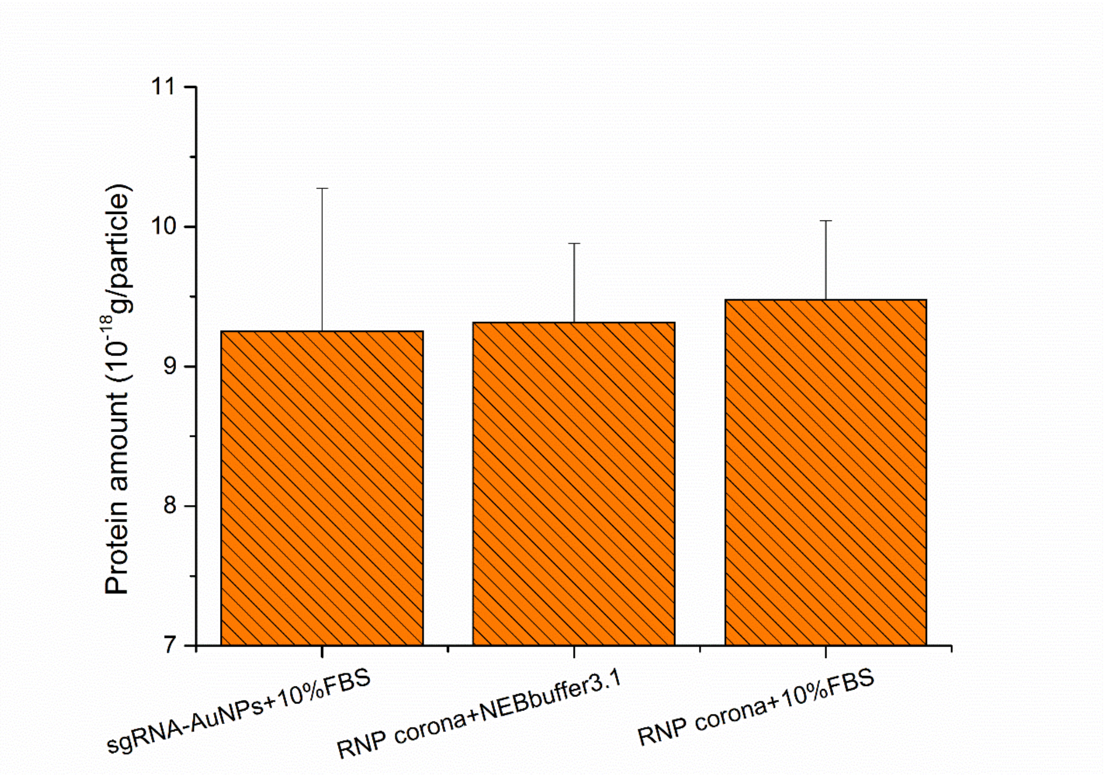

Figure S10. Comparison of non-specific protein coronae and binding-mediated RNP coronae (10:1) on their average amount of protein per nanoparticle (protein density). The sgRNA-AuNPs were incubated with 10% fetal bovine serum (FBS) for one hour. The RNP corona was incubated with 10% FBS or NEB buffer 3.1 for one hour. Each mixture was then centrifuged (21,000g, 30 min) and the particles washed for three times using 1×PBS buffer. The nanoparticles were incubated with the RIPA buffer (25 mM Tris-HCl pH 7.6, 150 mM NaCl, 1% NP-40, 1% sodium deoxycholate, 0.1% SDS) at room temperature for 30 min and then at 95 °C for 10 min. The amount of proteins released from nanoparticles was measured by using a Pierce™ bicinchoninic acid (BCA) protein assay kit (Thermofisher). Non-specific protein corona formed on sgRNA functionalized AuNPs (sgRNA-AuNPs) had a similar amount of protein as compared to the RNP corona.

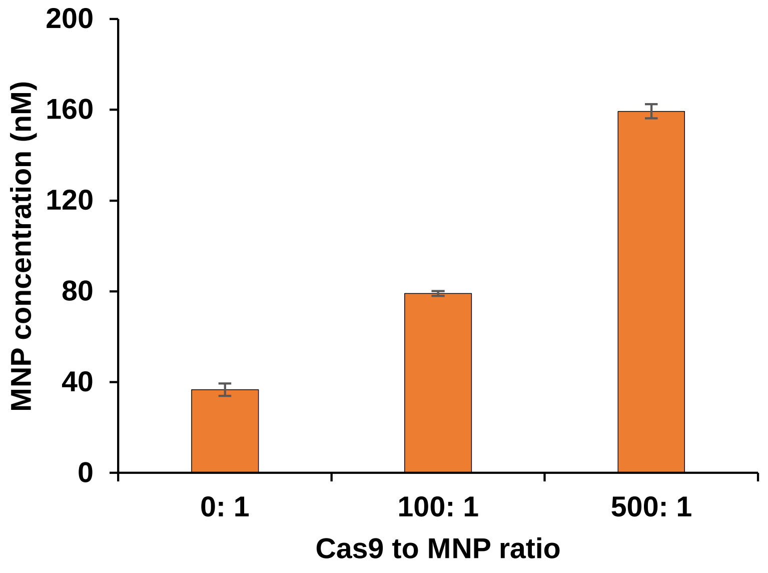

Figure S11. Uptake of the binding-mediated RNP corona formed using magnetic nanoparticles (MNPs) as the scaffold. Three preparations were compared: (1) the sgRNA functionalized MNP without loading of Cas9 (0:1), (2) the sgRNA functionalized MNP was incubated with 100-fold molar excess of Cas9, and (3) the sgRNA functionalized MNP was incubated with 500-fold molar excess of Cas9. A total of 1×10^5 Hela cells were incubated with 100 µL of 1 nM of the sgRNA-MNPs or the RNP-MNP corona for 4 h. After treatment, the cells were washed, detached, and digested with 20% HNO_3 at 60 °C overnight. The MNPs were determined by detecting Fe using ICP-MS.

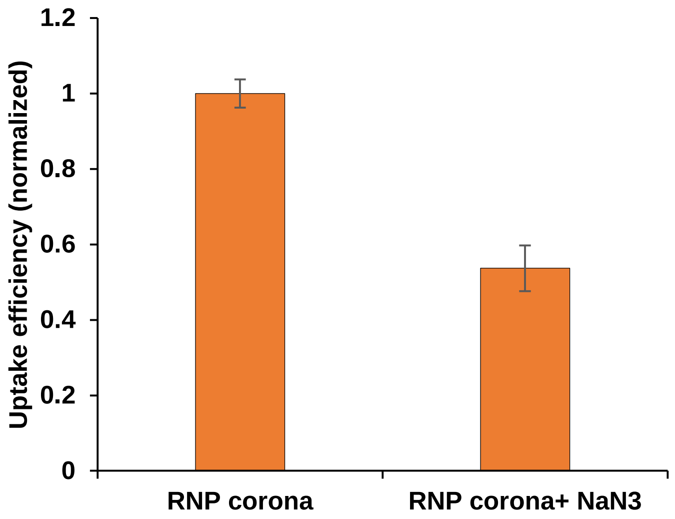

Figure S12. Cellular uptake of the binding-mediated RNP corona by Hela cells with and without the sodium azide pretreatment. One set of the Hela cells were pre-incubated with 50 nM NaN_3 for 1 h. Then, both sets of the Hela cells were incubated with 10 nM of the binding-mediated RNP corona (10:1) for 8 h. The intracellular concentrations of AuNPs were determined using inductively coupled plasma mass spectrometry (ICP-MS). The normalized uptake efficiency was compared on the basis of the intracellular concentrations of the RNP corona. The results show that the pretreatment with NaN_3 reduced cellular uptake of the RNP corona by ~46%.

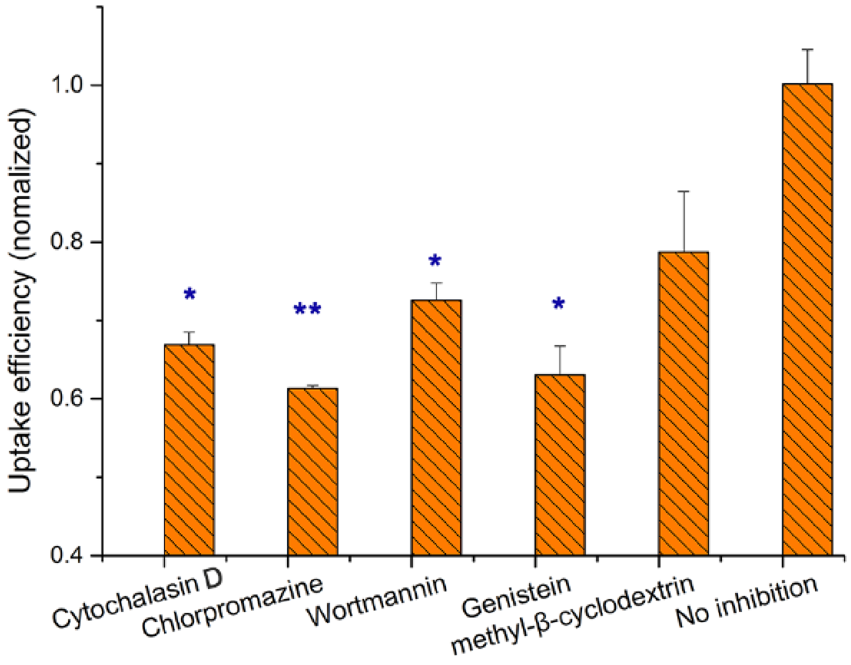

Figure S13. Cellular uptake of RNP corona affected by endocytosis inhibitors. Hela cells were either not incubated with an inhibitor (no inhibition) or pre-incubated for 30 min with 1 μM cytochalasin D, 5 μM chlorpromazine, 1 μM wortmannin, 100 μM genistein, and 2 mg/mL methyl-β-cyclodextrin, respectively. These endocytosis inhibitors were selected on the basis of previous work[2]. Then, the Hela cells were incubated with 10 nM of the binding-mediated RNP corona (10:1) for 8 h. The intracellular concentrations of AuNPs were determined using inductively coupled plasma mass spectrometry (ICP-MS). The normalized uptake efficiency was compared on the basis of the intracellular concentrations of the RNP corona. * denotes $p < 0.05$, and ** denotes $p < 0.01$. The pre-incubation of Hela cells with a clathrin-mediated endocytosis inhibitor (chlorpromazine), a caveolin-mediated endocytosis inhibitors (genistein), a phagocytosis inhibitor (cytochalasin D), and a receptor-mediated endocytosis inhibitor (wortmannin) resulted in significant decreases in cellular uptake of RNP corona. These results suggest that multiple endocytosis pathways are involved in the cellular uptake of RNP corona.

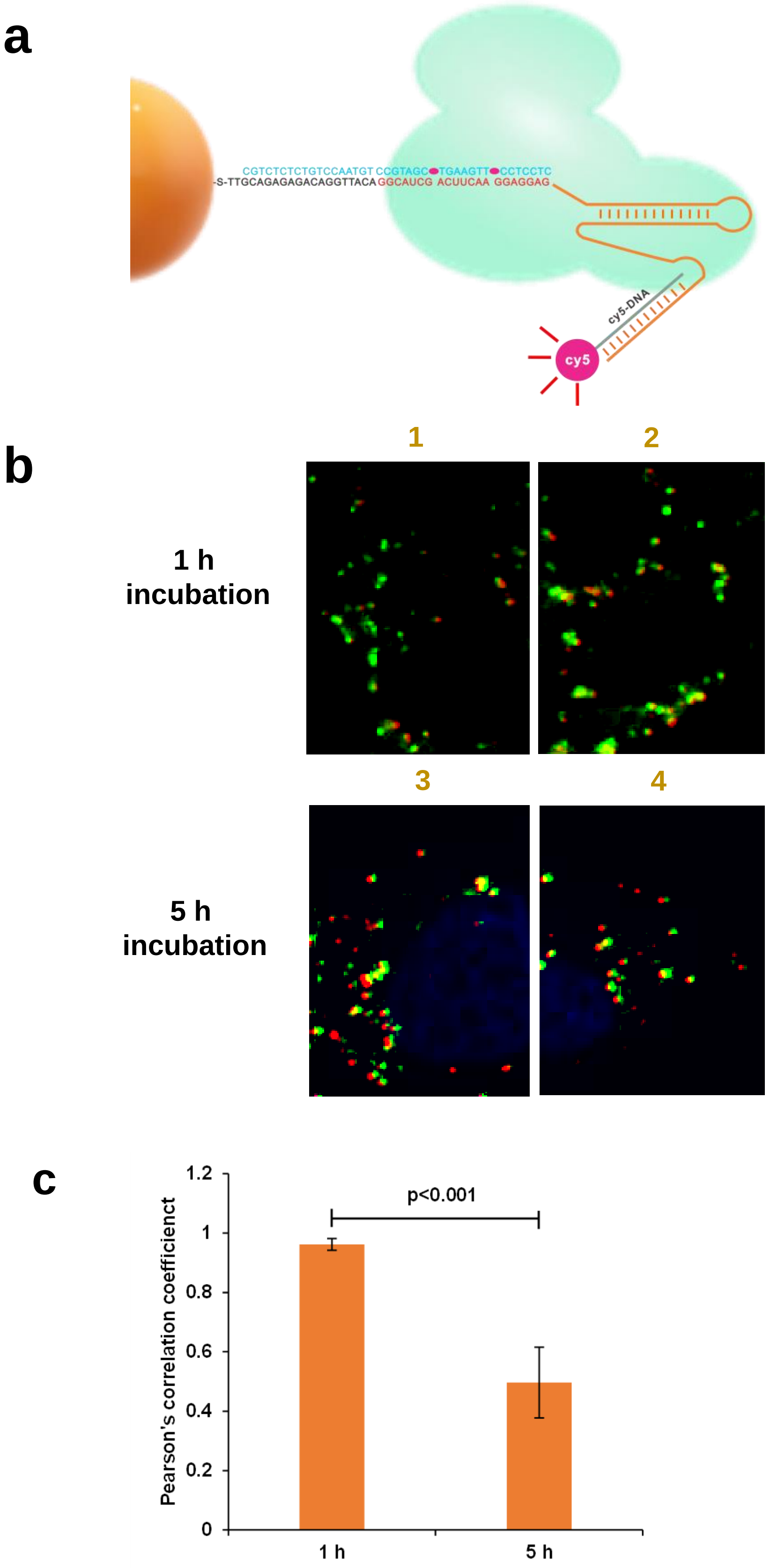

Figure S14. Endocytosis of the binding-mediated RNP corona. (a) One domain at the 3'-end of the sgRNA was hybridized with a Cy5-labeled complementary DNA (sequence of Cy5-26 as shown in Table S2). The fluorescent labeling of the sgRNA

complex with Cy5 was for tracking the uptake of the RNP coronae. This construct was used to generate the results shown in Figure 3. (b) Four zoomed-in areas from Figure 5, showing merged images from the detection of the RNP corona (red), LysoTracker Yellow HCK-123 (green, staining for endosomes/lysosomes), and DAPI (blue, staining for the nuclei). (c) Pearson's correlation coefficients showing the overlap between the RNP corona and endosomes. The ImageJ plugin colocalization Finder was used to process the images in (b) and calculate Pearson's correlation coefficients. A total of 40,000 Hela cells were seeded in an 8-well μ-slide one day for cell attachment. The cells were treated with the RNP corona (10:1) for 1 h and 5 h. Cells were washed and fluorescence images taken using confocal microscopy. The scale bar is 15 μm.

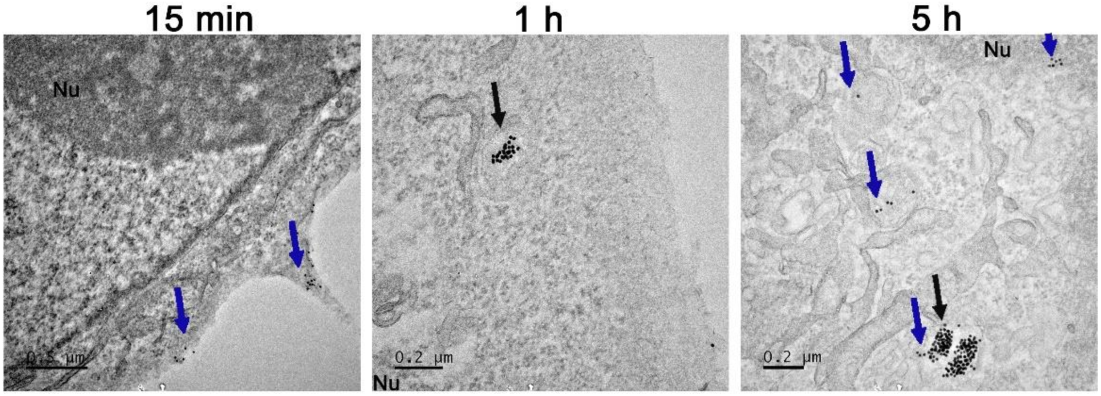

Figure S15. TEM images showing RNP corona in the cell over a 5-h time course of cell incubation with RNP corona. Cells were seeded on a Aclar sheet for 24 h, and then treated with RNP corona for 15 min, 1 h, and 5 h, respectively. The cells were washed with PBS for 5 times and fixed with 2% paraformaldehyde and 2.5% glutaraldehyde in 0.1 M phosphate buffer (PB). The fixed cells were then washed with 0.1M PB for 3 times, and post-fixed with 1% OsO_4 in 0.1 M PB for 1 hour at room temperature. After washed with water for 3 times, the cells were stained with 1%UA/H_2O at 4 ^oC and with lead aspartate solution at 60 ^oC, each for 15 min. The cells were then dehydrated with 30%, 50%, 70%, 95% and 100% ethanol, and infiltrated with 25%, 50%, 75% and 100% spurr's resin, which was polymerized at 70 ^oC. Ultrathin sections were prepared for imaging using JEOL 2100 Transmission Electron Microscope. The black arrows indicate RNP corona within endosomes/lysosomes and the blue arrows indicate RNP corona out of endosomes/lysosomes. A few RNP coronae were present at the cell membrane after 15 min incubation. After 1 h incubation, the RNP coronae were taken up and mostly co-localized with endosomes/lysosomes. Some RNP coronae escaped from endosomes/lysosomes after incubation for 5 h.

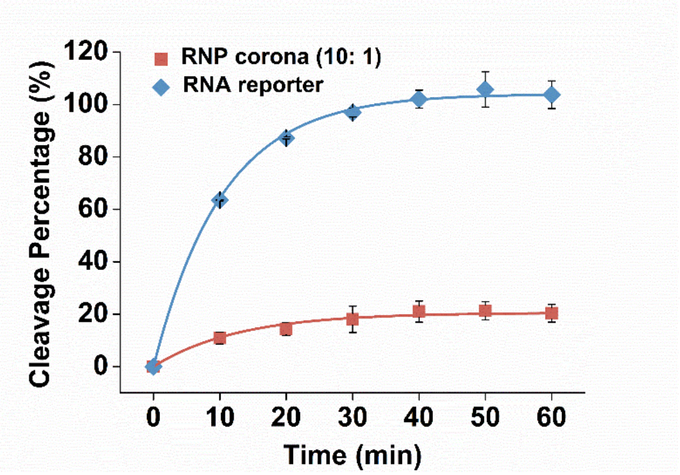

Figure S16. Enhanced stability of the binding-mediated RNP corona as compared to a RNA reporter. The sgRNA in the RNP corona was hybridized to a FAM-labeled DNA (FAM-26). The RNA reporter (RNaseAlert) was labeled with FAM and quencher on either end. The RNA reporter and the RNP corona (10:1) were each mixed with 10% fetal bovine serum (FBS). Cleavage of the RNA reporter and the sgRNA in the RNP corona was monitored over time by measuring the fluorescence intensity of FAM. The fluorescence of FAM was quenched in the intake RNA reporter and the intact RNP corona. These results show that the RNP corona protected the stability of the sgRNA.

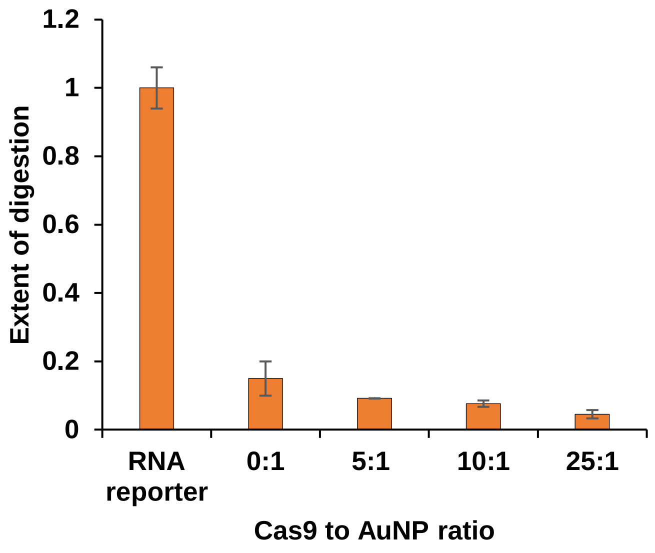

Figure S17. Protection of the sgRNA in the binding-mediated RNP coronae against digestion by RNase A. The sgRNA in the RNP coronae was hybridized to a FAM-labeled DNA (FAM-26). The RNA reporter (RNaseAlert), the sgRNA functionalized AuNPs (0:1), and the RNP coronae (5:1), (10:1) and (25:1) were each mixed with 1×PBS containing 70 micro-units (μunits) of RNase A. The intake RNA reporter and the intact RNP corona were not fluorescent because FAM was quenched in the intact molecules. Cleavage of the RNA reporter and the sgRNA in the RNP corona was monitored by measuring the fluorescence intensity of FAM. These results show that the RNP coronae protected the sgRNA from RNase digestion.

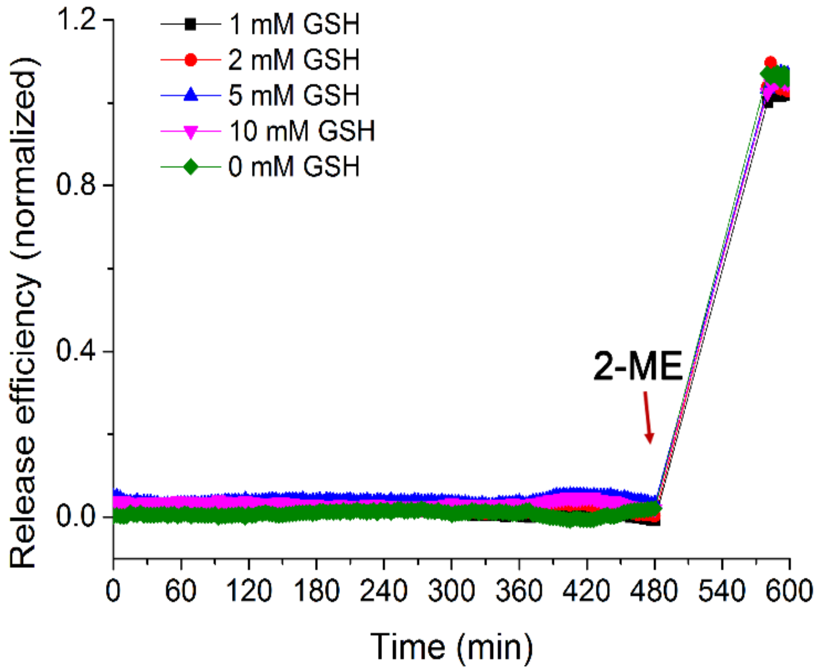

Figure S18. Stability of the binding-mediated RNP corona (BMPC) in the presence of glutathione (GSH). One nanomolar (1 nM) FAM-labeled BMPC (25: 1) was prepared in 100 µL of 25 mM Tris-HCl (7.4) containing 100 mM NaCl and 0, 1, 2, 5 or 10 mM GSH, respectively. These solutions were loaded onto a 96-well plate and the fluorescence was measured every 3 min for 8 h at room temperature. There was no fluorescence increase over time, indicating that the RNP corona was stable. Finally, 5 µL of 700 mM 2-mercaptoethanol (2-ME) was added into each solution to release FAM-labeled sgRNA from AuNPs. After 1 h incubation, fluorescence was monitored for another 20 min.

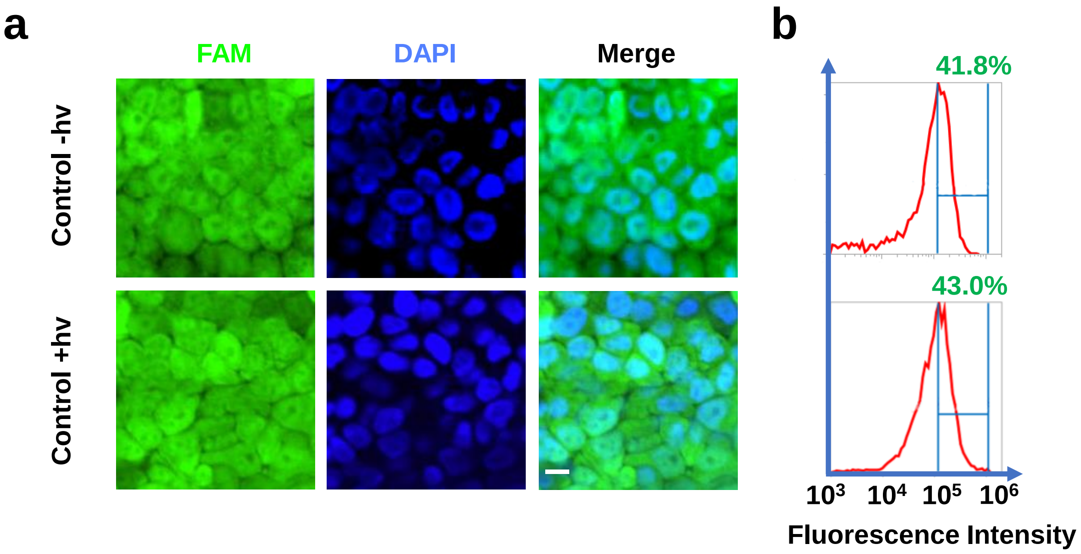

Figure S19. Confocal microscopy and flow cytometry monitoring of Hela-GFP cells. The cells were either UV irradiated (+hv) or not irradiated (–hv). (a) Confocal microscopy images of Hela-GFP cells that were either UV-irradiated (λ= 365 nm, 3.0 mW/cm^2) for 15 min or not irradiated. The scale bar is 10 µm. (b) Flow cytometry histograms of the Hela-GFP cells corresponding to treatment in (a).

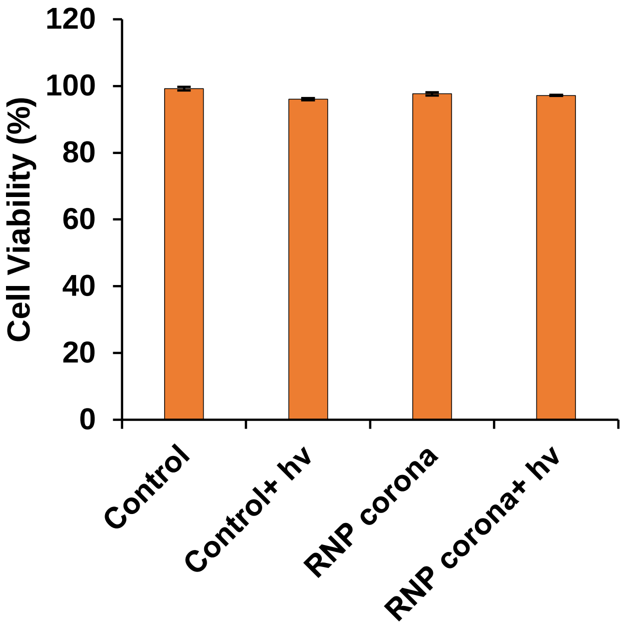

Figure S20. Viability of Hela cells after treatment with the RNP corona.

Supplementary References:

[1] C. H. J. Choi, L. Hao, S. P. Narayan, E. Auyeung, C. A. Mirkin, *Proc. Natl. Acad. Sci. U.S.A.* **2013**, *110*, 7625-7630.

[2] a) V. Francia, K. Yang, S. Deville, C. Reker-Smit, I. Nelissen, A. Salvati, *ACS Nano* **2019**, *13*, 11107-11121; b) H. Costa Verdera, J. J. Gitz-Francois, R. M. Schiffelers, P. Vader, *J. Control. Release* **2017**, *266*, 100-108. c) L.-H. Wang, K. G. Rothberg, R. G. W. Anderson, *J. Cell Biol*. **1993**, *123*, 1107-1117.
